# Single-Dose Intrathecal rAAV2-Retro Delivering Anti-hSOD1 miRNA Confers Robust Neuroprotection in Adult ALS Mice

**DOI:** 10.3390/cells15201890

**Published:** 2026-10-16

**Authors:** Xueqi Gong, Hao Wei, Lang Shen, Qin Wang, Jiayi Wu, Wenyuan Wang, Tonghui Xu

**Affiliations:** 1 Laboratory Animal Center, Fudan University, Shanghai 200433, China; 2 Laboratory Animal Resource Center, Fudan University, Shanghai 200433, China; 3 School of Life Sciences, Fudan University, Shanghai 200438, China; 4 Interdisciplinary Research Center on Biology and Chemistry, Shanghai Institute of Organic Chemistry, Chinese Academy of Sciences, Shanghai 200032, China; 5 Department of Rehabilitation Medicine, Huashan Hospital, Fudan University, Shanghai 200040, China

**Keywords:** amyotrophic lateral sclerosis (ALS), rAAV2-retro, miRNA, IGF1

## Abstract

Amyotrophic lateral sclerosis (ALS) kills within a few years of onset, and the drugs licensed for treatment change that course only marginally. Silencing *SOD1* has become a credible treatment route, but two gaps hold this approach back: an efficient vector platform for spinal cord transduction with limited peripheral transduction and evidence on whether adding neurotrophic support to gene silencing has any effect. We built an rAAV2-retro platform for intrathecal delivery. One intrathecal dose of an eGFP-expressing vector transduced all spinal cord segments and several brain regions, with a clear preference for spinal motor neurons and low expression in the liver and heart. Side by side in adult mice, we then used this platform to compare miRNA-mediated silencing of human *SOD1*, overexpression of *IGF1*, and the two together in *SOD1^G93A^* animals. A single injection of rAAV2-retro carrying an anti-*hSOD1* miRNA extended median survival by 25 days, improved motor performance, protected neuromuscular junctions, spared α-motor neurons, and damped gliosis and apoptosis. IGF1 on its own also lengthened survival and limited neuronal loss, but it left motor performance unchanged. The benefit of IGF1 over miRNA alone was observed on most endpoints under the conditions tested; a modest, nominally significant improvement in hindlimb clasping was observed, which should be interpreted as exploratory given the number of comparisons performed. Silencing of *hSOD1* therefore accounts for most of the benefit, and no statistically detectable additional benefit was observed for IGF1 on top of hSOD1 silencing under the conditions tested. A single intrathecal dose of rAAV2-retro-miRNA is thus an effective gene silencing approach in the *SOD1^G93A^* mouse model, with limited peripheral transduction in the liver and heart observed in C57BL/6J mice. However, these data also show the difficulty of achieving additional benefit by adding IGF1 under the conditions tested.

## 1. Introduction

Amyotrophic lateral sclerosis (ALS) destroys motor neurons in the motor cortex, brainstem, and spinal cord, and it does so selectively, leaving most other neuronal populations intact. Weakness, muscle wasting, and spasticity spread relentlessly until patients reach paralysis and respiratory failure; median survival is 2–5 years [1,2] from symptom onset. Incidence is roughly 1–2 per 100,000 person-years, and prevalence is 6–9 per 100,000, with onset peaking in the seventh decade [3,4,5]. No single lesion explains the disease: protein aggregation, oxidative stress, mitochondrial failure, glutamate excitotoxicity, and sustained neuroinflammation all contribute, and each amplifies the others [6,7,8,9,10]. About 10% of ALS cases are familial (fALS); the other 90% are sporadic (sALS) [7]. Within fALS, *SOD1* was the first gene to be implicated and still accounts for roughly 20% of familial cases; more than 200 variants have now been cataloged [10,11]. The mechanism is a toxic gain of function rather than a loss of dismutase activity: mutant protein misfolds and accumulates. Evidence of abnormal SOD1 folding extends beyond inherited SOD1-associated disease, with similar changes reported in a proportion of sporadic ALS cases [12]. Available disease-modifying therapies, including riluzole, edaravone, and sodium phenylbutyrate–taurursodiol, offer only limited clinical benefit and do not directly target the underlying genetic causes of the disease [11,13,14]. Closing that gap in SOD1-associated ALS means intervening directly on *SOD1*.

Suppressing *SOD1* with RNA interference (RNAi) or antisense oligonucleotides (ASOs) is effective in preclinical models of SOD1-ALS [15,16,17]. FDA approval of tofersen for *SOD1*-ALS has carried the principle into the clinic and established *SOD1* as a tractable target in patients [18,19,20]. Tofersen, however, must be given repeatedly via lumbar puncture, which burdens patients, produces swings in cerebrospinal fluid (CSF) exposure, and carries procedural risk. Adeno-associated virus (AAV) vectors offer the alternative of durable transgene expression in the central nervous system from one administration. However, AAV9 brings its own problem: it escapes the CSF into the circulation and accumulates in peripheral tissue—above all, the liver—where immune activation and hepatotoxicity can compromise safety and efficacy [21]. A capsid that can efficiently transduce the spinal cord with minimal peripheral leakage would be advantageous for such an approach.

Insulin-like growth factor 1 (IGF1) has broad neurotrophic activity, keeping motor neurons alive, supporting axonal growth, and protecting muscle [22,23]. Preclinical studies have shown that *IGF1* overexpression confers therapeutic benefit in ALS models, although its overall efficacy remains modest [24,25,26]. IGF1 and *SOD1* silencing operate through distinct pathways, raising the possibility that their combination might yield synergistic effects. However, whether adding IGF1 to *SOD1* silencing yields benefits beyond *SOD1* silencing alone has not been systematically evaluated.

Therefore, we devised a combination strategy that integrates *SOD1* gene silencing with IGF1-mediated neurotrophic support delivered by rAAV2-retro, an AAV variant engineered for retrograde transport and efficient spinal cord transduction. It carries a cell-penetrating peptide in the AAV2 capsid, which provides it with efficient retrograde axonal transport [27]. Previous studies have shown that rAAV2-retro mediates strong retrograde transduction after intramuscular injection and, when administered peripherally in neonatal mice, efficiently transduces spinal motor neurons [28]. In our parallel study, we achieved widespread spinal transduction and significant therapeutic benefits in neonatal *SOD1^G93A^* mice with intramuscular injection of rAAV2-retro expressing an artificial miRNA targeting human *SOD1* (*hSOD1*) [29]. However, in adult animals the efficiency of retrograde transduction via peripheral routes was markedly reduced [30]. We reasoned that placing rAAV2-retro directly in the cerebrospinal fluid would sidestep those peripheral barriers and restore efficient, widespread transduction of spinal motor neurons in adult animals. We therefore constructed rAAV2-retro vectors carrying an artificial miRNA targeting *hSOD1* and/or an *IGF1* overexpression cassette. As the RNA interference effector, we chose an artificial microRNA (miRNA), which offers a more favorable safety profile than conventional short hairpin RNA approaches [31,32].

To test this hypothesis, four vectors (scramble control, IGF1 alone, miRNA alone, and combination) were administered as a single intrathecal injection to adult *SOD1^G93A^* mice. We then systematically evaluated disease progression, motor function, survival, and multiple neuropathological parameters across treatment groups to compare *SOD1* silencing alone with its combination with *IGF1* overexpression.

## 2. Materials and Methods

### 2.1. Animals

C57BL/6J mice (Jackson Laboratory, stock no. 000664) and *SOD1^G93A^* transgenic mice carrying multiple copies of the human mutant *SOD1^G93A^* transgene (Jackson Laboratory, stock no. 004435) were used in this study; the latter served as the ALS mouse model. All mice were obtained from Jackson Laboratory (JAX, Bar Harbor, ME, USA). Genotyping was performed to identify offspring carrying the high-copy human mutant *SOD1^G93A^* transgene, and male and female transgenic offspring were randomly assigned to the indicated experimental groups.

Random allocation was applied during animal grouping, and blinding was introduced whenever feasible during experimental evaluation to minimize potential bias. Mice were maintained in a specific pathogen-free facility at Fudan University (Shanghai, China) under controlled environmental conditions. All animal experiments received approval from the Institutional Animal Care and Use Committee of Fudan University and were conducted according to the institutional guidelines for laboratory animal care and use. For survival and behavioral analyses, group sizes were as follows: scramble, *n* = 12; IGF1 alone, *n* = 8; miRNA alone, *n* = 8; combination, *n* = 13. Male and female mice were used in each group (scramble, 6 females and 6 males; IGF1 alone, 4 females and 4 males; miRNA alone, 4 females and 4 males; combination, 6 females and 7 males), and randomization was not stratified by sex. For histopathological analyses, *n* = 5 mice per group were used.

### 2.2. Design and Construction of rAAV2-Retro Expression Vectors

A series of recombinant AAV expression vectors were constructed based on the rAAV2-retro backbone to enable both exogenous protein expression and RNA interference functions. Each vector contained a CAG promoter-driven dual-functional expression cassette incorporating a protein-coding element and an RNA regulatory element. The protein-coding components consisted of either the eGFP reporter gene or the human *IGF1* coding sequence, while the RNA regulatory components included either a *hSOD1*-targeting artificial miRNA sequence or a scramble miRNA control sequence according to the experimental design.

All recombinant AAV genomes were flanked by AAV2 inverted terminal repeats (ITRs) and incorporated regulatory elements, including the woodchuck hepatitis virus post-transcriptional regulatory element (WPRE) and the SV40 polyadenylation signal (SV40 polyA), to enhance transcript stability and maintain transgene expression. Four rAAV2-retro vectors were generated: rAAV2-retro-CAG-eGFP-Scramble, rAAV2-retro-CAG-IGF1-Scramble, rAAV2-retro-CAG-eGFP-miRNA, and rAAV2-retro-CAG-IGF1-miRNA, which were subsequently used for viral production and in vivo functional evaluation.

For the *hSOD1* RNA interference module, the artificial miRNA sequence was designed based on the *hSOD1* transcript, with the recognition site located within exon 2. The miRNA sequence was incorporated into a miR-30a-derived scaffold to support intracellular miRNA processing. A scramble sequence was designed as a negative control and showed no significant complementarity to known human or mouse transcripts.

The human *IGF1* coding sequence used in this study was derived from a previously reported study [33]. The artificial miRNA and scramble sequences are provided below, with the miRNA guide strand indicated by underlining: 5′-TGTTTGAATGAGGCTTCAGTACTTTACAGAATCGTTGCCTGCACATCTTGGAAACACTTGCTGGGATTACTTCTTAGGTTAACCCAACAGAAGGCAAGAAGGTATATTGCTGTGACATGAGCGTCATATGGAATCCATGTATGAGCCACAGATGTAAAGGTGGATGGAAAGTATGTACTGCCTCGACTTCAAGGTACTTTACAATTATCTGTTTACTACTTACTTATCTCTGATCATTTTTACAGCTGAATTAAAATGTATAAATTAAATCACTTTA-3′.

### 2.3. rAAV2-Retro Vector Production and Titration

Recombinant rAAV2-retro particles were generated in HEK293 cells using a triple-plasmid production system. This system consisted of an ITR-flanked transfer plasmid carrying the expression cassette; a packaging plasmid encoding AAV2 Rep proteins and the engineered rAAV2-retro capsid; and the adenoviral helper plasmid pHELP. Following collection and purification, viral genome titers were determined via qPCR and expressed as genome copies per milliliter GC/mL.

### 2.4. Intrathecal Injection

Intrathecal delivery was carried out according to a previously established protocol [34,35]. Mice were anesthetized with isoflurane using an animal anesthetic machine, and injection began only once the pedal withdrawal reflex had ceased. The lumbar region was shaved beforehand. Vector (2 × 10^11^ GC per mouse) was delivered into the intrathecal space at L4–L5 through a 30-G needle. A tail flick or brief hindlimb movement confirmed entry into the cerebrospinal fluid. To limit backflow, the needle was held in position for 5 min after injection before being withdrawn slowly. Mice recovered in a warmed cage and were watched until spontaneous activity returned. At the designated endpoint, mice were deeply anesthetized with isoflurane and perfused transcardially, first with phosphate-buffered saline (PBS) to clear blood.

### 2.5. Body Weight Measurement

Body weight was monitored weekly at a fixed time point from one week after intrathecal injection until the endpoint. Measurements were performed using a calibrated electronic balance with a precision of 0.1 g.

### 2.6. Behavioral Analysis

Behavioral assessments were initiated one week after intrathecal injection. All behavioral tests were performed at a fixed time point during the light cycle to minimize the potential influence of circadian variation. During the study, mice were assigned random identification numbers, and behavioral testing and data analysis were conducted in a blinded manner with respect to experimental group allocation to reduce observer bias.

Rotarod Test. Motor coordination and balance were assessed using an accelerating rotarod ( KW-6D, KEW BASIS, Nanjing, China), as previously described with modifications [26]. The rod speed was increased from 3 to 40 rpm within 1 min. Each mouse was tested three times with intervals of at least 15 min, and the mean latency to fall was calculated for statistical analysis. Mice completing more than two passive rotations while holding onto the rod were recorded as falls.

Limb Clasping Assay. Mice were suspended by the tail for 10 s with the head facing downward, and hindlimb clasping behavior was evaluated. A score was assigned when the clasping posture was maintained for more than 2 s. Hindlimb clasping was graded on a 0–3 scale according to previously published criteria [36].

Grip-Strength Test. A grip strength meter equipped with a horizontal grid (KEW BASIS, KW-ZL, Nanjing, China) was used to measure forelimb and hindlimb strength separately, as previously described [16]. The tail was gently pulled backward while the body was kept horizontal until the mouse released its grip. The maximum grip force was automatically recorded by the instrument. Each mouse underwent three trials separated by at least 1 min, and the mean value was used as the final grip strength and expressed in grams.

### 2.7. Disease Onset and Survival Evaluation

Disease onset was defined as a 10% reduction in body weight from the peak body weight, a widely used and objective indicator of disease progression in *SOD1^G93A^* mice [37]. The humane endpoint was determined when mice exhibited complete bilateral hindlimb paralysis and were unable to right themselves within 30 s after being placed on their back.

### 2.8. Compound Muscle Action Potential (CMAP) Measurement

Following anesthesia, stimulating electrodes were placed at the sciatic notch, while the recording electrode was inserted into the muscle belly of the ipsilateral gastrocnemius muscle. The reference electrode was positioned at the tendon, and the ground electrode was placed on the tail. A single supramaximal electrical stimulus was applied to evoke CMAP responses. The CMAP amplitude (measured from the baseline to the negative peak) and latency (from stimulus onset to the onset of the negative peak) were recorded [38]. During the measurement, body temperature was maintained at a constant level to ensure accurate assessment of neuromuscular conduction.

### 2.9. Tissue Preparation and Sectioning

Brain, spinal cord, and liver tissues were post-fixed in 4% PFA at 4 °C for 24 h and subsequently cryoprotected in 20% and 30% sucrose solutions sequentially, each for 24 h. The tissues were then sectioned at 30 μm thickness using a cryostat (CM1950, Leica Biosystems, Wetzlar, Germany). Half of the gastrocnemius muscle samples were dehydrated through a graded ethanol series, paraffin-embedded, and sectioned at 3 μm thickness using a rotary microtome (CM1950, Leica Biosystems, Wetzlar, Germany). The remaining gastrocnemius muscle was dissected under a stereomicroscope into small bundles containing 3–4 muscle fibers and processed for neuromuscular junction (NMJ) staining.

### 2.10. Western Blot

Tissue samples were lysed in RIPA buffer (20101ES60, YEASEN, Shanghai, China) supplemented with a protease inhibitor cocktail (MCE, HY-K0010, Monmouth Junction, NJ, USA) for protein extraction. Protein concentrations were determined using a BCA assay kit (A55860, Thermo Fisher Scientific, Waltham, MA, USA). Equal amounts of protein were mixed with loading buffer, denatured by boiling, separated by SDS-PAGE, and transferred onto PVDF membranes. After blocking, membranes were incubated with the indicated primary antibodies at 4 °C overnight, followed by thorough washing with TBST and incubation with corresponding HRP-conjugated secondary antibodies at room temperature. After the final wash, immunoreactive bands were visualized with an enhanced chemiluminescence (ECL) substrate and documented using a chemiluminescence imaging system. Protein abundance was determined by densitometric analysis in ImageJ and expressed relative to β-Actin.

The antibodies used were as follows: eGFP (sc-5384, Santa Cruz Biotechnology, Dallas, TX, USA), β-Actin (ab8226, Abcam, Cambridge, UK), hSOD1 (sc-17767, Santa Cruz Biotechnology, Dallas, TX, USA), and goat anti-mouse IgG conjugated to HRP (A0216, Beyotime, Shanghai, China).

### 2.11. ELISA

Lumbar spinal cord was collected and homogenized on ice without delay. The homogenate was clarified by centrifugation, and the supernatant was used for ELISA. Human IGF1 was measured by enzyme-linked immunosorbent assay (ELISA) with a commercial kit (ab100545, Abcam, Cambridge, UK) following the manufacturer’s instructions.

### 2.12. Immunofluorescent Staining

Frozen sections were washed with PBS, followed by permeabilization with 0.3% Triton X-100 (85111, Thermo Fisher Scientific, Waltham, MA, USA) and blocking with 5% bovine serum albumin (BSA) (A2153, Merck, Darmstadt, Germany) to reduce nonspecific antibody binding. The sections were incubated with the corresponding primary antibodies at 4 °C overnight. After thorough washing with PBS, sections were incubated with fluorescently labeled secondary antibodies at room temperature in the dark. Following secondary antibody incubation, sections were washed again and mounted with an antifade mounting medium containing DAPI. The stained sections were imaged using a slide scanner (PANNORAMIC 250 Flash III, 3DHISTECH, Budapest, Hungary) and a Leica confocal microscope (sp8, Leica Biosystems, Wetzlar, Germany).

For NMJ staining, muscle bundles were washed with PBS and blocked for 1 h at room temperature in PBS containing 5% goat serum and 0.5% Triton X-100 (85111, Thermo Fisher Scientific, Waltham, MA, USA). The samples were incubated with an anti-neurofilament (NF) primary antibody at 4 °C overnight to label presynaptic axons, followed by incubation with fluorescent secondary antibodies and fluorophore-conjugated α-bungarotoxin (α-BTX, 00018, Biotium, Fremont, CA, USA) at room temperature in the dark for 2 h to label postsynaptic acetylcholine receptors. After washing, the samples were mounted and imaged using a spinning-disk microscope. NMJs were scored as innervated when NF-positive axon terminals overlapped with α-BTX-positive endplates.

The antibodies applied for immunofluorescence experiments were as follows. Primary antibodies against eGFP (ab184601, Abcam, Cambridge, UK), ChAT (ab178850, Abcam, Cambridge, UK), GFAP (ab7260, Abcam, Cambridge, UK), NeuN (ABN91, Millipore, Burlington, MA, USA), Iba1 (ab178846, Abcam, Cambridge, UK), hSOD1 (2770, Cell Signaling Technology, Danvers, MA, USA), and neurofilament heavy polypeptide (NF-H; ab4680, Abcam, Cambridge, UK) were used. Fluorescent secondary antibodies included Alexa Fluor 488-conjugated goat anti-mouse IgG (ab150113, Abcam, Cambridge, UK), Alexa Fluor 555-conjugated goat anti-rabbit IgG (ab150078, Abcam, Cambridge, UK), and Alexa Fluor 647-conjugated goat anti-chicken IgG (ab150171, Abcam, Cambridge, UK).

### 2.13. TUNEL Staining

The Beyotime One-Step TUNEL Apoptosis Assay Kit (C1090, Beyotime, Shanghai, China) was used following the manufacturer’s instructions. The stained sections were imaged using a 3DHISTECH slide scanner (PANNORAMIC 250 Flash III, 3DHISTECH, Budapest, Hungary) and a Leica confocal microscope (sp8, Leica Biosystems, Wetzlar, Germany), respectively.

### 2.14. HE Staining

Paraffin-embedded sections were dewaxed and rehydrated through a graded ethanol series (A500737, Sangon Biotech, Shanghai, China). The sections were then sequentially stained with hematoxylin (E607317-0100, Sangon Biotech, Shanghai, China) and eosin (G1120, Solarbio, Beijing, China). After staining, sections were dehydrated through graded ethanol, cleared in xylene, and mounted with neutral resin. Images of the stained sections were acquired using a 3DHISTECH slide scanner (PANNORAMIC 250 Flash III, 3DHISTECH, Budapest, Hungary).

### 2.15. Image Quantification and Analysis

For all histological analyses, the experimental unit was the individual mouse. Multiple subsamples per animal (NMJs, muscle fibers, cells, sections, or ROIs) were first averaged to generate a single animal-level summary measure, which was then used for statistical comparison.

Quantification of Motoneuron and TUNEL-Positive Cells. Images acquired using a slide scanner were imported into QuPath 0.5.0, where positive cells in the target channels were identified by optimizing detection parameters, followed by manual verification. The number of motoneurons was determined based on the quantification of cells showing ChAT and NeuN double positivity.

Quantification of Glial and hSOD1 Signal Intensity. Raw fluorescence images were imported into ImageJ v1.53, and individual channels were separated for analysis. The target channel was selected, background signals were subtracted, and regions of interest (ROIs) were manually delineated to measure the mean fluorescence intensity.

Analysis of NMJs. ImageJ was used to process the Z-stack images following the published protocol [35]. Based on the extent of overlap between presynaptic and postsynaptic components, NMJs were classified into four categories: fully innervated (complete overlap between presynaptic and postsynaptic signals), partially innervated (presynaptic coverage >50% but <100%), partially denervated (presynaptic coverage >0% but ≤50%), and completely denervated (presence of postsynaptic structures with complete absence of presynaptic signals). At least 50 NMJs per animal were randomly selected and scored in a blinded manner.

Muscle Cross-Sectional Area (CSA) Analysis. Images of HE-stained gastrocnemius cross-sections acquired by slide scanning were imported into ImageJ. Individual muscle fibers were identified based on their morphology and boundaries, and the contours of each fiber were manually outlined. The CSA of each muscle fiber was then measured.

### 2.16. Statistical Analyses

Statistical analyses were conducted using GraphPad Prism 10. Data are presented as means ± SEM for continuous variables. For the molecular and histological datasets, normality was assessed using the Shapiro–Wilk test and variance homogeneity using Levene’s test. Both assumptions were met, and one-way ANOVA followed by Tukey’s post hoc test was used for these datasets. Survival analysis was pre-specified as the principal confirmatory endpoint; survival outcomes were visualized by Kaplan–Meier curves and compared using the log-rank (Mantel–Cox) test. All other endpoints were analyzed as exploratory, and *p* values for these endpoints were not adjusted for multiplicity across the full family of endpoints. For behavioral outcomes, a cross-sectional comparison at week 19 was performed using one-way ANOVA followed by Tukey’s post hoc test. Week 19 was selected as the last time point at which all four groups still had measurable animals for behavioral assessment (forelimb grip: 5; hindlimb grip: 5; rotarod: 4); at week 20, fewer than 3 animals per group remained measurable for all three behavioral tests. Mixed-effects models were not used because the missing data at late time points were not missing at random, as they resulted from disease progression and animals reaching humane endpoints. This limits the validity of conventional longitudinal inference. As limb clasping scores did not meet the assumptions of normality and variance homogeneity, the Kruskal–Wallis test was used, followed by Dunn’s post hoc test. Statistical significance was assigned at *p* < 0.05, with thresholds indicated as * *p* < 0.05, ** *p* < 0.01, *** *p* < 0.001, and **** *p* < 0.0001.

## 3. Results

### 3.1. Intrathecal Delivery of rAAV2-Retro Mediates Widespread CNS Transduction with Limited Peripheral Off-Target Expression

**Figure 1:**
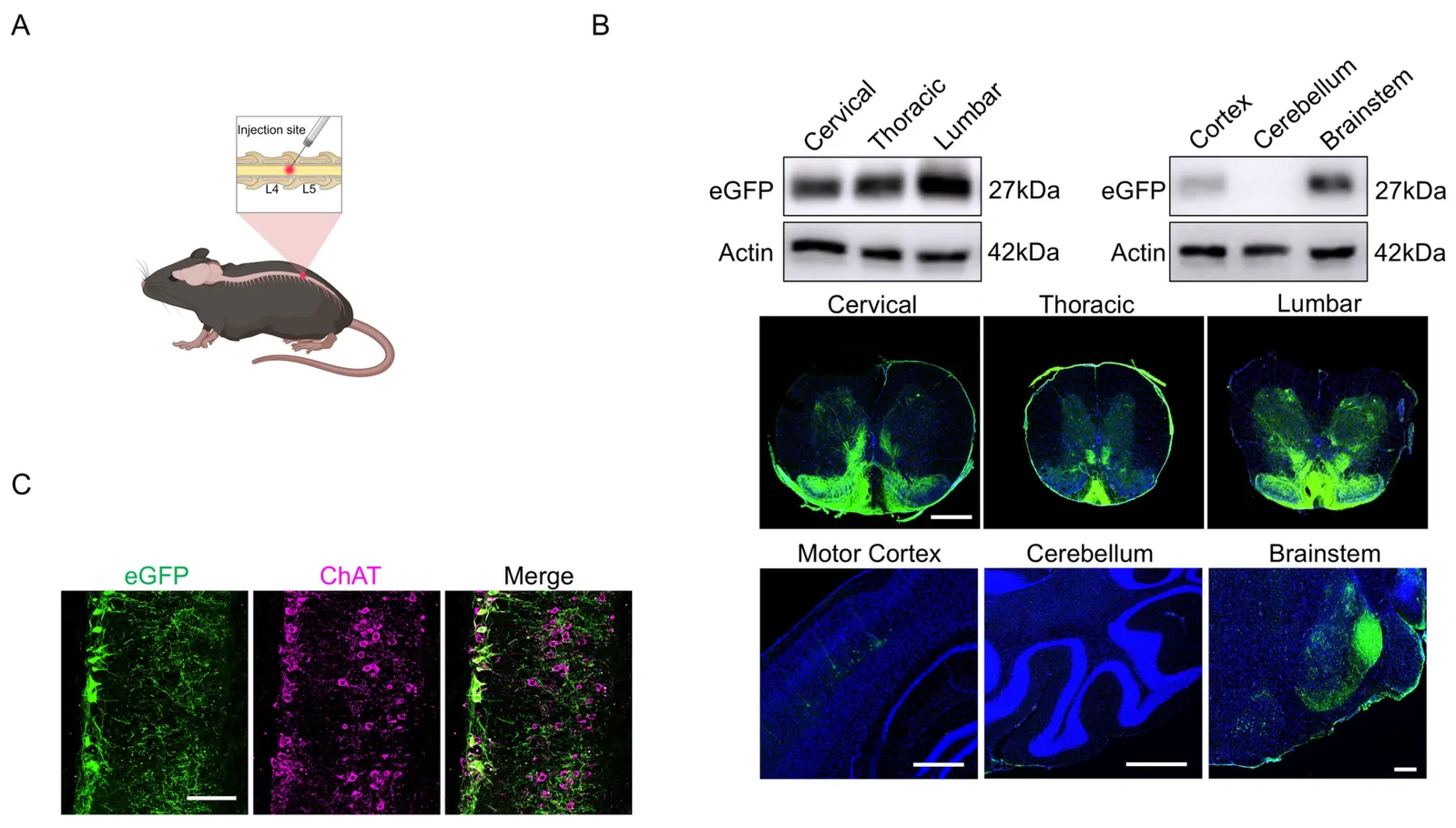
Transduction of spinal cord and brain after a single intrathecal AAV injection. (**A**) Schematic of the intrathecal (IT) route. The virus entered through the intervertebral foramen into the cerebrospinal fluid at the indicated lumbar (L) position of the spinal cord. (**B**) eGFP expression following IT injection of rAAV2-retro-CAG-eGFP (2 × 10^11^ GC per mouse) in adult mice. Upper panel: Immunoblotting analysis of eGFP expression in tissue homogenates from indicated spinal cord segments (cervical, thoracic, lumbar), as well as brain regions, including motor cortex, cerebellum, and brainstem. Lower panel: Representative images of different spinal cord and brain regions. Scale bars, 200 μm. (**C**) eGFP signal in the lumbar spinal cord of adult mice 4 weeks after injection. Longitudinal sections were co-stained with anti-ChAT antibody to label spinal motor neurons. Scale bars, 200 μm.

To evaluate the transduction efficiency and biodistribution of rAAV2-retro following intrathecal (IT) administration, we generated a CAG-eGFP reporter vector and injected a single dose of 2 × 10^11^ GC per mouse into the cerebrospinal fluid via the lumbar intervertebral foramen (Figure 1A). Four weeks later, strong eGFP expression was detected throughout the spinal cord (Figure 1B). Immunoblotting showed the highest eGFP levels in the lumbar cord, with lower but clearly detectable expression in the cervical and thoracic segments and in the brainstem. Expression was also detected in the motor cortex, though it was weaker than in the spinal cord or brainstem. Immunofluorescence showed that almost every eGFP-positive cell in the lumbar spinal cord co-localized with choline acetyltransferase (ChAT) (Figure 1C). The strong eGFP signal in the posterior column reflects labeled fibers within the ascending sensory tracts, whereas the co-localization of eGFP with ChAT in the anterior horn confirms motor neuron tropism. Quantification of eGFP and ChAT co-localization across spinal cord segments showed that 95.4 ± 1.72% of eGFP-positive cells in the cervical segment, 94.08 ± 1.34% in the thoracic segment, and 96.2 ± 1.30% in the lumbar segment were ChAT-positive (specificity). Conversely, 53.35 ± 1.87% of ChAT-positive motor neurons in the cervical segment, 49.60 ± 1.79% in the thoracic segment, and 54.98 ± 2.66% in the lumbar segment were eGFP-positive (coverage) (*n* = 4 mice per segment, Figure S1). Notably, compared with the widely used AAV9 vector delivered via the same intrathecal route, rAAV2-retro showed substantially lower transduction in the liver and heart (Figure S2), consistent with a potentially favorable biodistribution profile. The liver and heart were examined as the principal peripheral organs susceptible to AAV9 leakage [39]. In addition, rAAV2-retro showed robust transduction across the spinal cord, with a segmental distribution pattern showing prominent enrichment in the lumbar region, comparable to that observed with AAV9 (Figure S3). Intrathecal rAAV2-retro therefore reaches the CNS broadly while largely sparing peripheral tissue, which is the required profile for a gene-delivery vector for ALS.

### 3.2. rAAV2-Retro Vectors Achieve Efficient IGF1 Overexpression and hSOD1 Silencing In Vivo

**Figure 2:**
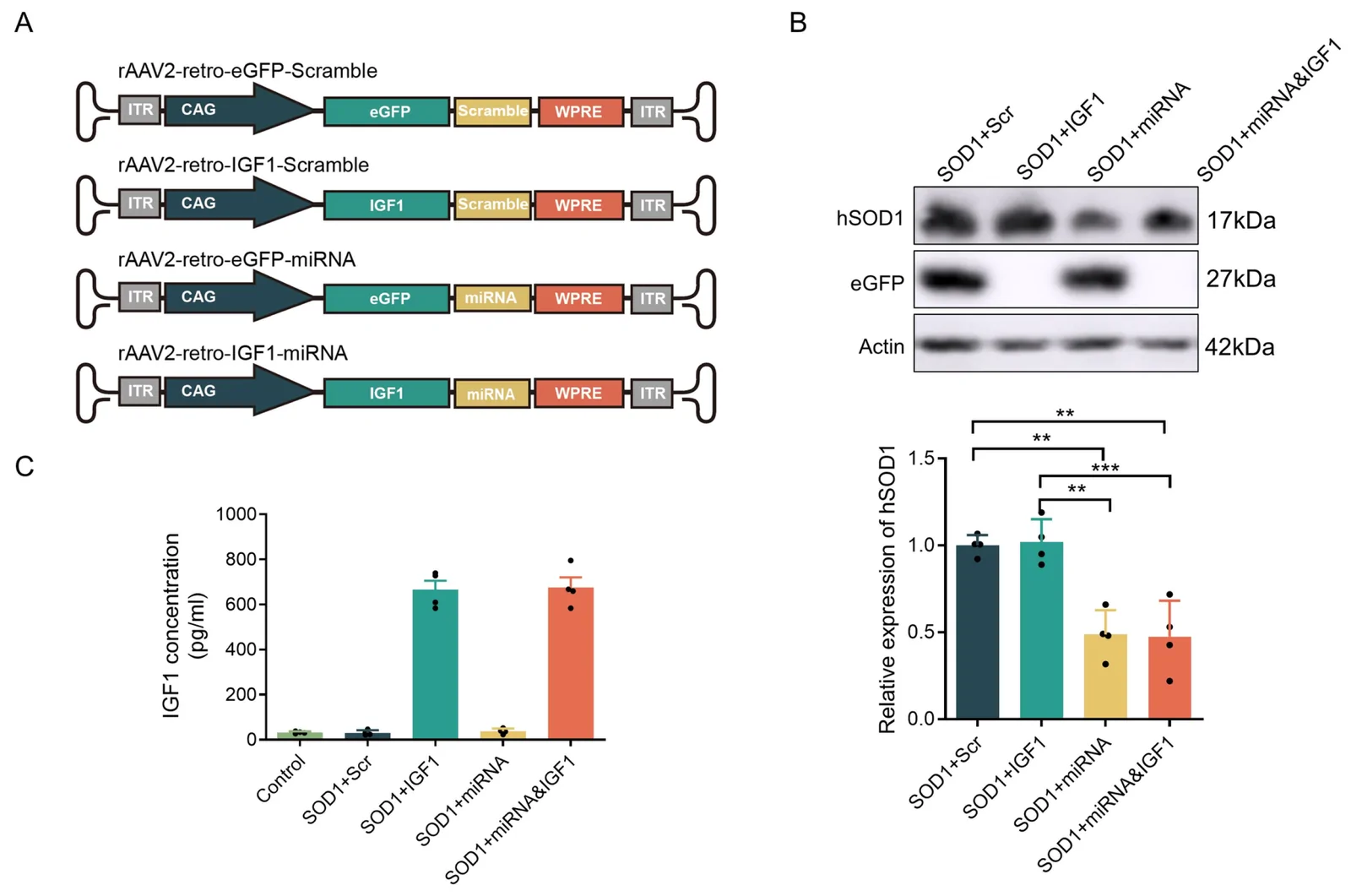
Design and in vivo validation of viral tools. (**A**) Construct design of rAAV2-retro-eGFP-Scramble, rAAV2-retro-IGF1-Scramble, rAAV2-retro-eGFP-miRNA, and rAAV2-retro-IGF1-miRNA vectors; promoter and expression elements sit between ITRs. (**B**) Upper panel: Western blot analysis of lumbar spinal cord samples from SOD1 mice administered with the indicated vectors to assess *hSOD1* expression. Lower panel: Densitometric quantification of hSOD1 protein levels normalized to β-actin. One-way ANOVA with Tukey’s post hoc test. (**C**) ELISA analysis of IGF1 protein levels in the lumbar spinal cord of SOD1 mice treated with the indicated AAV vectors. Mice were injected intrathecally at 8 weeks of age (2 × 10^11^ GC per mouse), and tissues were collected 4 weeks post-injection. Data are means ± SEM, *n* = 4 mice per group. ANOVA with Tukey’s post hoc test. ** *p* < 0.01, *** *p* < 0.001.

Knocking down mutant *hSOD1* by RNA interference is an established route to treatment in *SOD1*-linked ALS [40,41]. Given that IGF1-conditioned microglia have been reported to limit motor neuron apoptosis in *SOD1*^G93A^ mice [24], we asked whether adding *IGF1* overexpression to miRNA-mediated knockdown of *hSOD1* would be more beneficial than either on its own. We therefore constructed four rAAV2-retro vectors: rAAV2-retro-CAG-eGFP-Scramble (scramble), rAAV2-retro-CAG-IGF1-Scramble (IGF1 alone), rAAV2-retro-CAG-eGFP-miRNA (miRNA alone), and rAAV2-retro-CAG-IGF1-miRNA (combination) (Figure 2A). To confirm that the vectors did what they were designed to do—that is, knock down *hSOD1* and raise *IGF1* in the spinal cord of SOD1 mice—we injected them intrathecally into 8-week-old *SOD1*^G93A^ mice at a dose of 2 × 10^11^ GC per mouse. Four weeks later, Western blotting showed that hSOD1 protein levels in the lumbar spinal cord were significantly lower in mice given the miRNA-alone or combination vectors than in scrambles (Figure 2B). Densitometric quantification showed that hSOD1 protein levels were reduced by 51.3% in the miRNA-alone group and 52.6% in the combination group relative to the scramble control (Figure 2B, lower panel; Table 1). ELISA confirmed a large rise in *IGF1* in the IGF1-alone and combination groups (Figure 2C). A single intrathecal dose of rAAV2-retro-based vectors thus delivers both efficient *hSOD1* silencing and sustained *IGF1* expression in the spinal cord of SOD1 mice.

### 3.3. miRNA-Mediated hSOD1 Knockdown Extends Survival in SOD1 Mice

**Figure 3:**
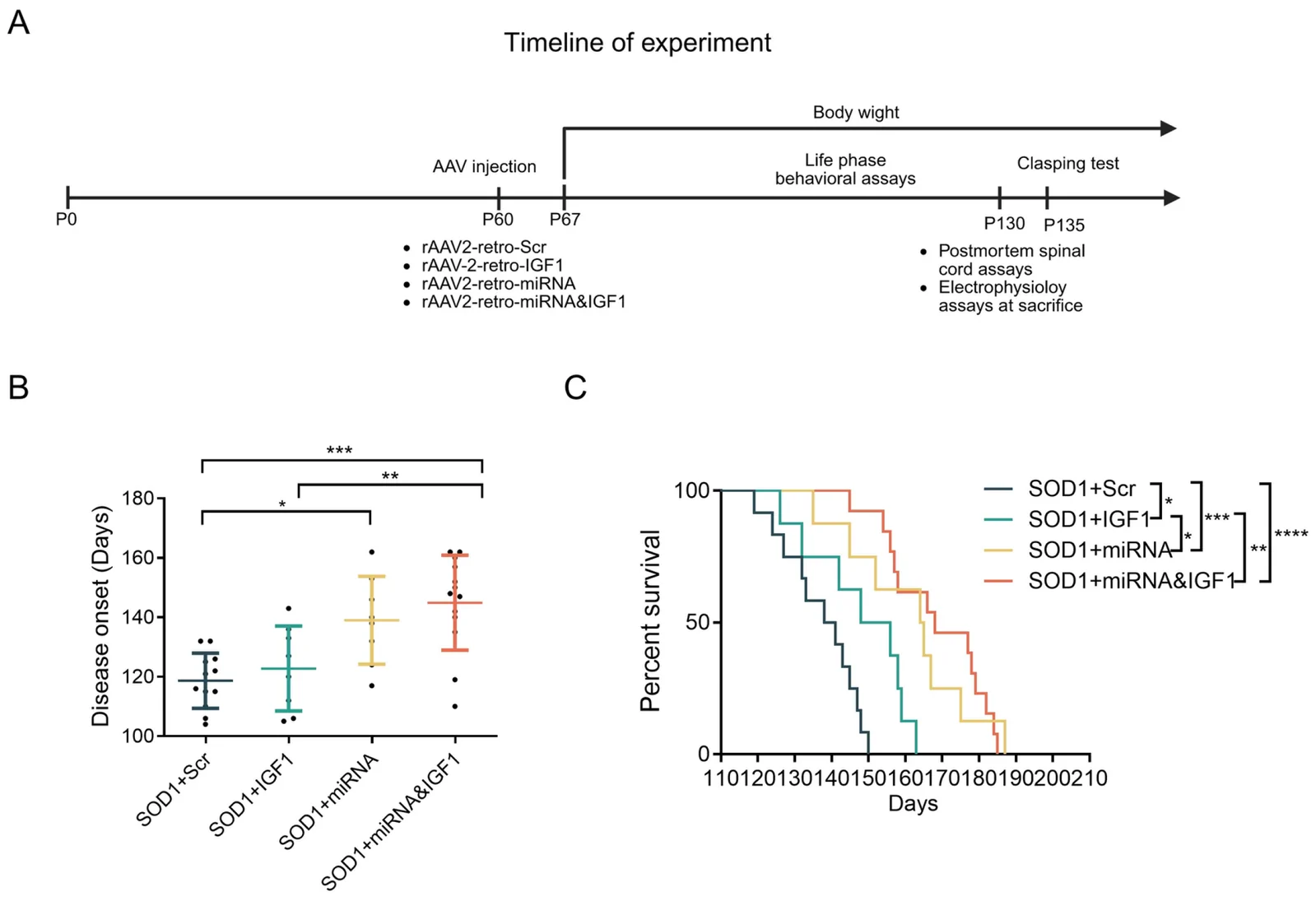
Assessment of disease onset, disease duration, and survival in SOD1 mice following intrathecal delivery of rAAV2-retro-based vectors. (**A**) Experimental timeline and the ante-mortem and post-mortem analyses performed. P, postnatal day. (**B**) Comparison of disease onset among SOD1 mice receiving different rAAV2-retro vector treatments. Data represent means ± SEM. One-way ANOVA with Tukey’s post hoc test. (**C**) Kaplan–Meier survival curves of SOD1 mice in each treatment group. Survival log-rank (Mantel–Cox) test (scramble *n* = 12; IGF1 alone *n* = 8; miRNA alone *n* = 8; combination *n* = 13). * *p* < 0.05, ** *p* < 0.01, *** *p* < 0.001, **** *p* < 0.0001.

Figure 3A sets out the timeline. All mice were injected intrathecally at postnatal day 60, and behavioral testing began one week later, comprising body weight, rotarod performance, and grip strength (scramble *n* = 12; IGF1 alone *n* = 8; miRNA alone *n* = 8; combination *n* = 13). Mice designated for histopathological analysis were euthanized at day 130 (*n* = 5 per group). Onset was again scored at a 10% fall from peak weight. Compared with scramble mice, the miRNA-alone and combination group showed a significant delay in disease onset (Figure 3B; Table 1). Notably, compared with untreated scramble mice (median survival, 139.5 days), the IGF1-alone, miRNA-alone, and combination groups demonstrated significantly prolonged median survival, extending to 152 days (+9.0%; log-rank χ^2^ = 5.79, *p* = 0.0161), 164.5 days (+17.9%; log-rank χ^2^ = 12.65, *p* = 0.0004), and 168 days (+20.4%; log-rank χ^2^ = 19.82, *p* < 0.0001), respectively (Figure 3C). Survival in the miRNA-alone and combination groups did not differ statistically (Table 1), though it was consistently below the combination curve (Figure 3C). Pairwise comparisons confirmed that combination therapy significantly outperformed IGF1 alone in median survival but did not differ significantly from miRNA alone (Table 1). Survival gain in this model is therefore mainly attributable to miRNA-mediated *hSOD1* silencing; the extra contribution from IGF1 did not reach statistical significance in this cohort.

### 3.4. miRNA-Based Therapies Preserve Motor Function in SOD1 Mice

**Figure 4:**
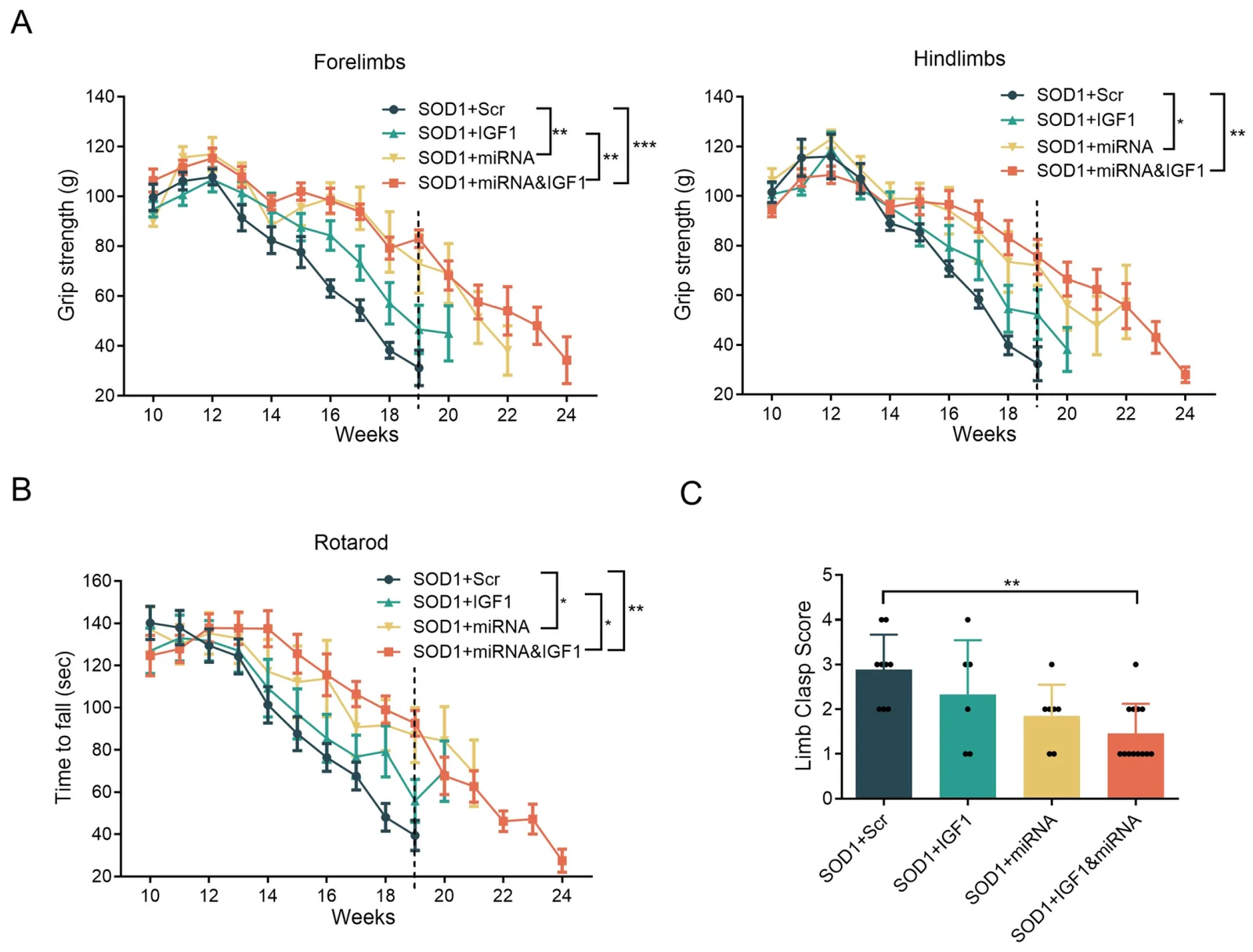
Motor function in SOD1 mice following intrathecal delivery of rAAV2-retro-based vectors. (**A**) Forelimb and hindlimb grip strength in SOD1 mice treated with different rAAV2-retro vectors. (**B**) Rotarod performance of SOD1 mice in the indicated treatment groups. (**C**) Limb clasping scores in SOD1 mice treated with the indicated vectors. (**A**,**B**) Data are means ± SEM; one-way ANOVA with Tukey’s post hoc test at week 19. (**C**) Data are means ± SEM; Kruskal–Wallis test followed by Dunn’s post hoc test (scramble *n* = 12; IGF1 alone *n* = 8; miRNA alone *n* = 8; combination *n* = 13). * *p* < 0.05, ** *p* < 0.01, *** *p* < 0.001.

Motor performance was gauged by forelimb and hindlimb grip strength, rotarod latency, and limb clasping score. Compared with scrambles, the IGF1-alone group showed no significant improvement in any of these measures (Table 1). In contrast, the miRNA-alone group showed significantly improved forelimb and hindlimb grip strength and rotarod performance, although limb clasping scores were not significantly reduced relative to controls (Table 1). The combination (IGF1 + miRNA) group showed the most robust and comprehensive benefits, with significantly better performance across all four motor parameters: increased forelimb and hindlimb grip strength, prolonged rotarod latency, and reduced limb clasping scores (Figure 4; Table 1). The combination and miRNA-alone groups did not differ significantly on any motor measure (Table 1); the functional gain observed with the combination was largely attributable to *hSOD1* silencing.

### 3.5. miRNA-Based Therapies Protect Neuromuscular Junction Integrity and Transmission

**Figure 5:**
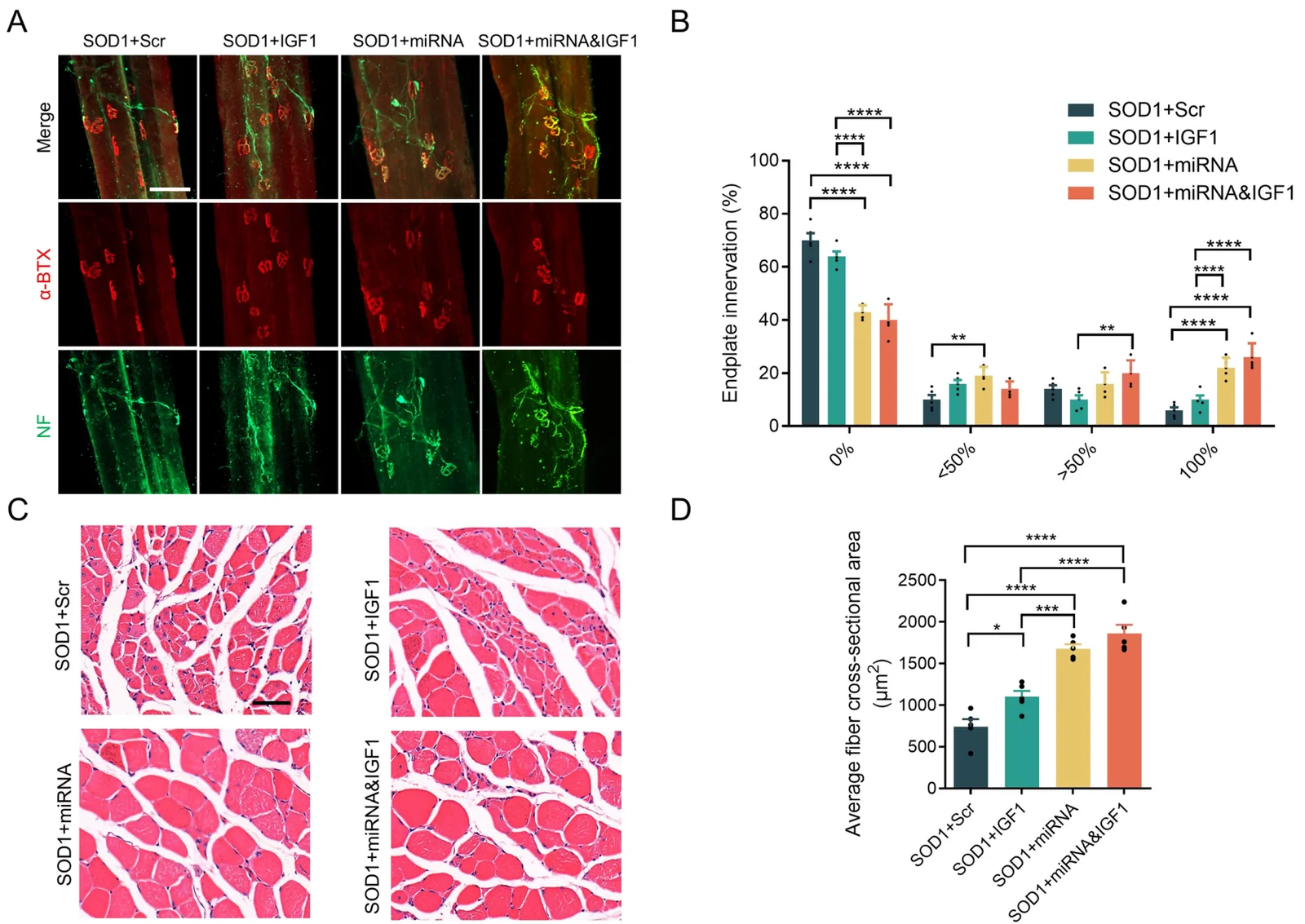
Neuromuscular junction integrity and muscle fiber morphology in SOD1 mice following intrathecal delivery of rAAV2-retro-based vectors. (**A**) NMJ morphology was examined in gastrocnemius muscle sections from SOD1 mice receiving the indicated rAAV2-retro vectors. Presynaptic axons were visualized by neurofilament (NF) staining and postsynaptic acetylcholine receptors by α-bungarotoxin (α-BTX). NF-labeled axon terminals overlapping with α-BTX-positive endplates were scored as innervated NMJs. Scale bar, 100 μm. (**B**) Presynaptic coverage was used to classify NMJs as fully innervated (100%), partially innervated (>50%), partially denervated (≤50%), or completely denervated (0%). (**C**) Histological assessment of gastrocnemius muscle by HE staining. Scale bar, 50 μm. (**D**) Quantitative analysis of average muscle fiber cross-sectional area (CSA). Data represent means ± SEM. One-way ANOVA with Tukey’s post hoc test; *n* = 5 mice per group. Multiple NMJs and muscle fibers per animal were averaged to generate a single value per mouse before statistical analysis. * *p* < 0.05, ** *p* < 0.01, *** *p* < 0.001, **** *p* < 0.0001.

**Figure 6:**
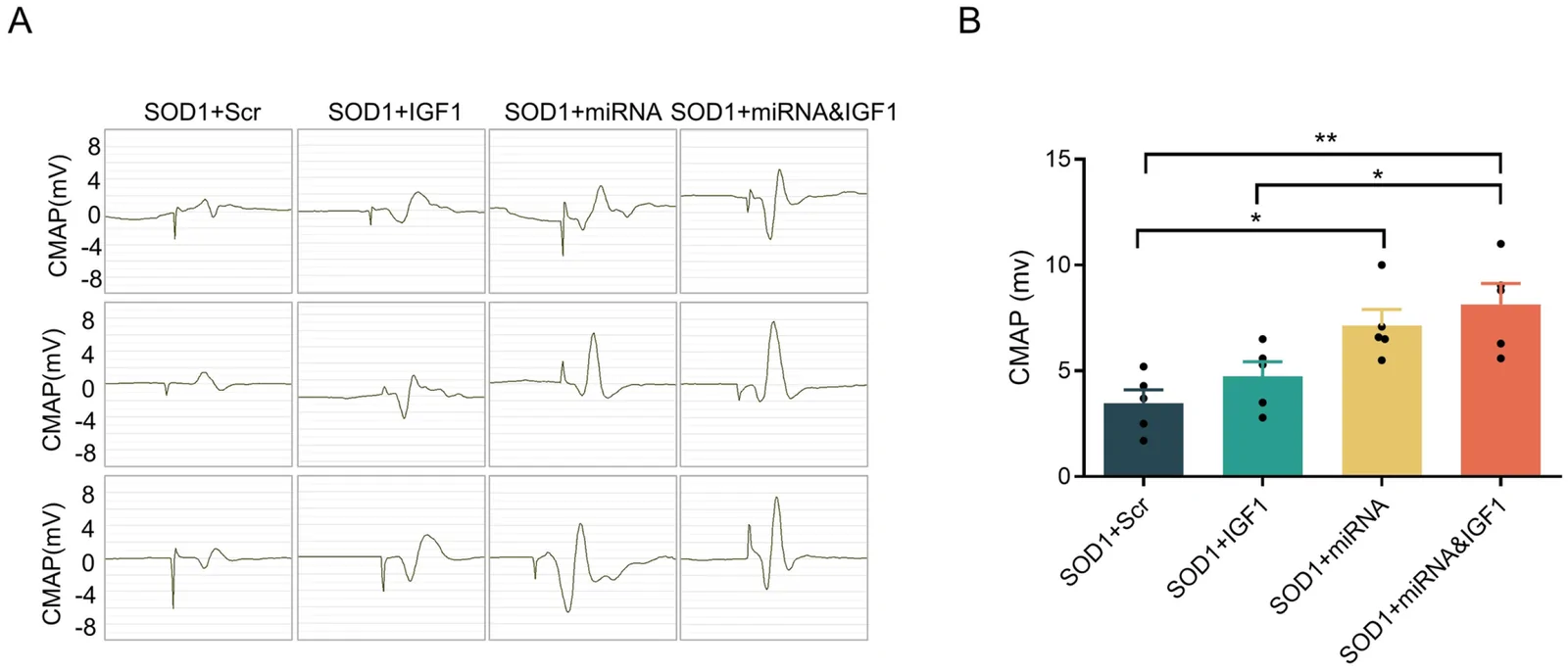
Assessment of neuromuscular transmission in SOD1 mice following intrathecal delivery of rAAV2-retro-based vectors. (**A**) Representative compound muscle action potential (CMAP) recordings. Each row shows traces from an individual mouse. (**B**) Quantitative analysis of CMAP amplitudes. Data represent means ± SEM, *n* = 5 mice per group. One-way ANOVA with Tukey’s post hoc test. Multiple CMAP recordings per animal were averaged to generate a single value per mouse before statistical analysis. * *p* < 0.05, ** *p* < 0.01.

By day 130, only 7.6% of neuromuscular junctions (NMJs) in the scramble mice remained fully innervated; the rest were partly or wholly denervated (Figure 5A,B). Notably, in *SOD1*^G93A^ mice treated with rAAV2-retro-CAG-eGFP-miRNA (miRNA alone) or rAAV2-retro-CAG-IGF1-miRNA (combination), the proportion of innervated NMJs markedly increased, and that of denervated NMJs markedly decreased (Figure 5A,B). Quantification of the mean cross-sectional area of gastrocnemius muscle fibers showed that the IGF1-alone, miRNA-alone, and combination treatments all significantly attenuated muscle atrophy relative to scrambles (Figure 5C,D; Table 1). Notably, the miRNA-alone and combination treatments conferred the most robust protection, with effect sizes exceeding those of IGF1 alone (Table 1). We also recorded compound muscle action potential (CMAP) amplitudes as a measure of neuromuscular transmission. Both the miRNA-alone and combination treatments raised CMAP amplitude significantly above scrambles, whereas IGF1 alone did not (Figure 6; Table 1). Neither muscle fiber area nor CMAP amplitude separated the miRNA-alone group from the combination group (Table 1).

### 3.6. miRNA-Based Therapies Rescue α-Motor Neurons and Suppress Neuroinflammation

**Figure 7:**
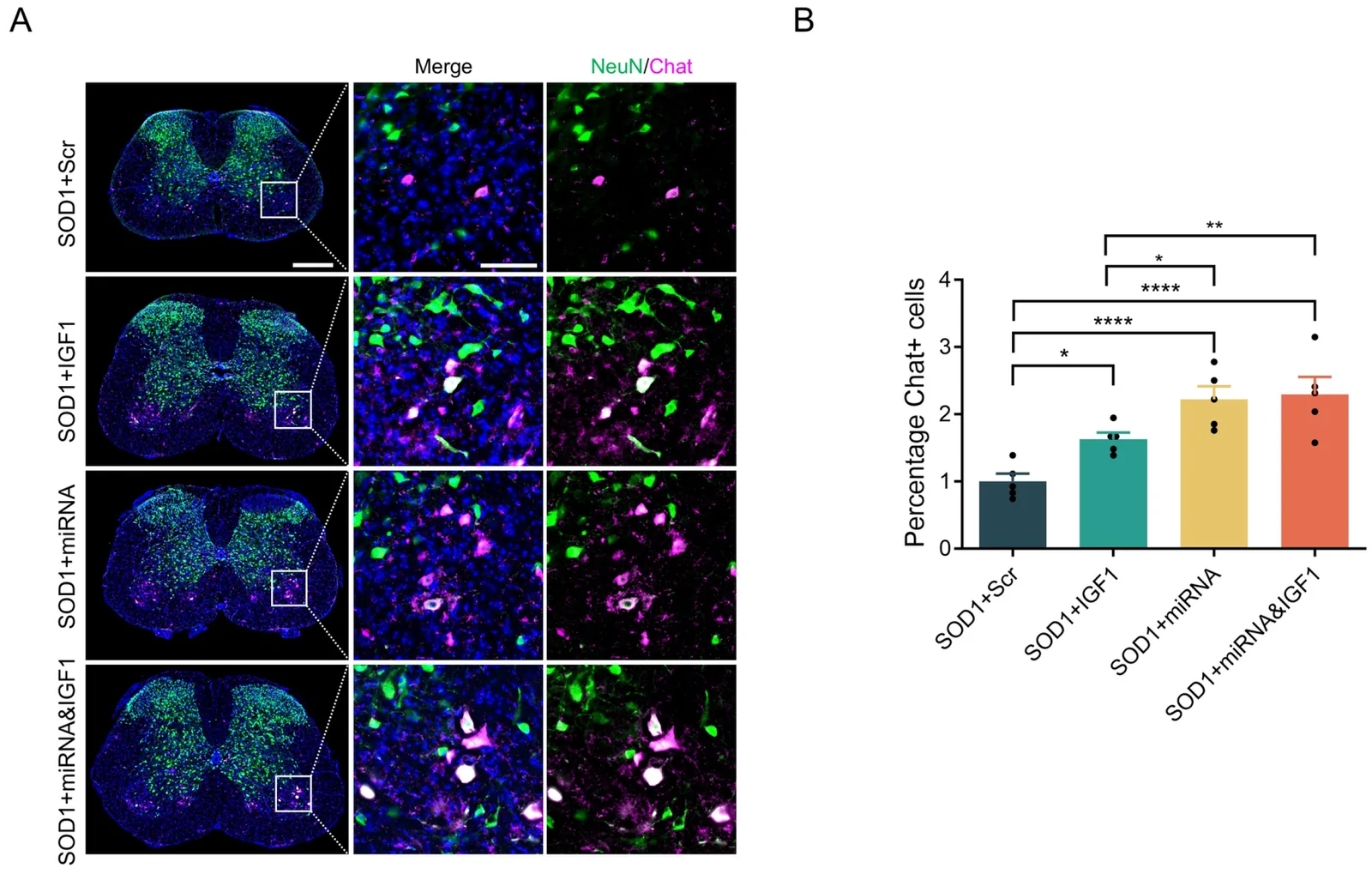
Survival of α-motor neurons in the spinal cord of SOD1 mice following intrathecal delivery of rAAV2-retro-based vectors. (**A**) Lumbar spinal cord immunofluorescence in SOD1 mice following administration of different rAAV2-retro constructs. Color coding: NeuN, green; ChAT, magenta; DAPI, blue. Scale bars, 400 µm and 100 µm. (**B**) α-motor neuron counts in lumbar spinal cord regions. Data represent means ± SEM. One-way ANOVA with Tukey’s post hoc test; *n* = 5 mice per group. Multiple spinal cord sections per animal were averaged to generate a single value per mouse before statistical analysis. * *p* < 0.05, ** *p* < 0.01, **** *p* < 0.0001.

Immunohistochemical analysis at day 130 revealed that α-motor neuron numbers in the lumbar spinal cord were lowest in the scramble group. The IGF1 alone, miRNA-alone, and combination treatments all significantly increased α-motor neuron survival relative to scrambles (Figure 7A,B; Table 1). Pairwise comparisons further indicated that the miRNA-alone and combination treatments did not differ significantly in their neuroprotective effects, and no statistically detectable additional benefit of IGF1 was observed within combination therapy under the conditions tested (Table 1).

**Figure 8:**
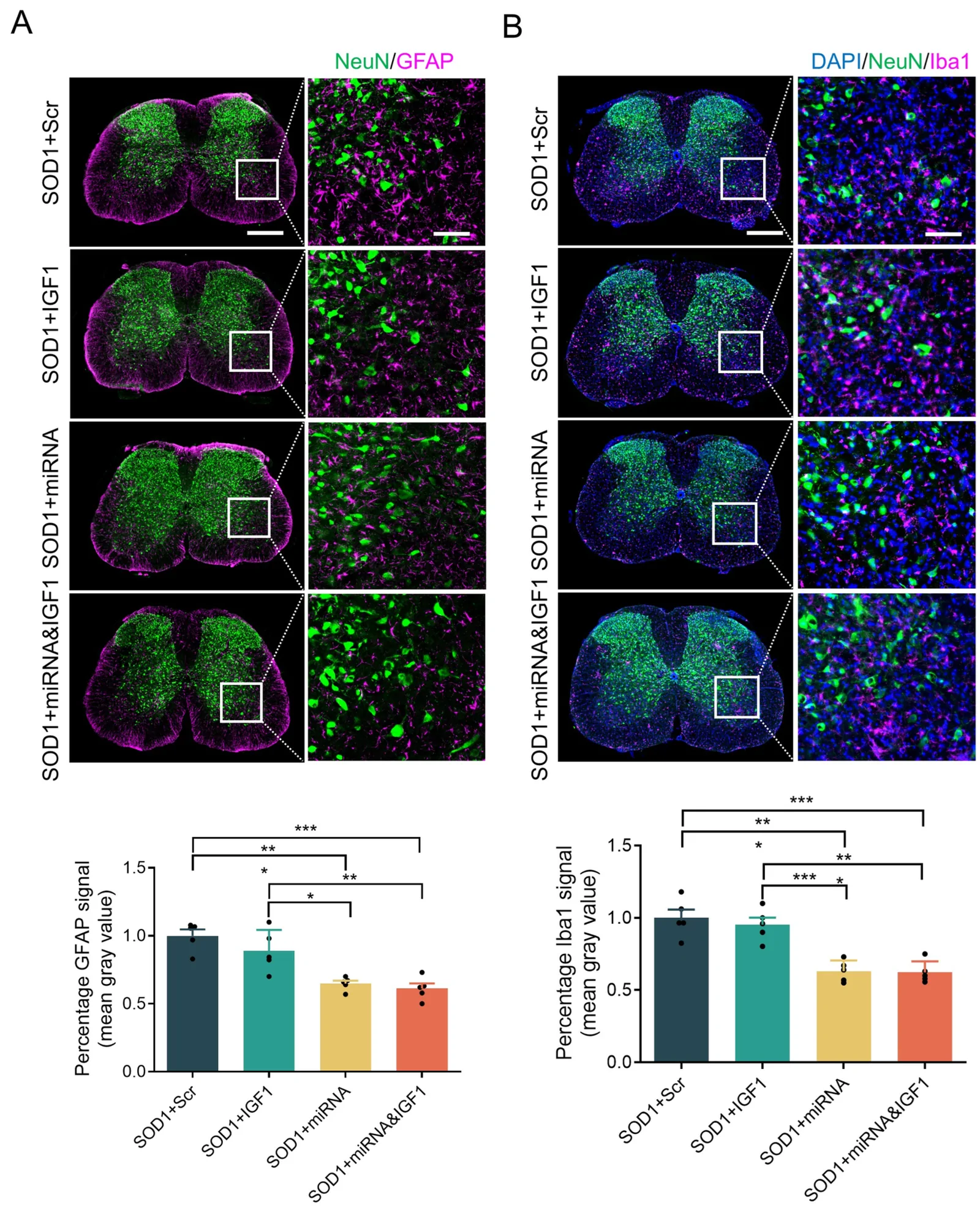
Neuroinflammation in the spinal cord of SOD1 mice following intrathecal delivery of rAAV2-retro-based vectors. (**A**) GFAP immunofluorescence analysis in lumbar spinal cord sections from SOD1 mice receiving the indicated rAAV2-retro vectors. Representative images are shown in the upper panels, and GFAP fluorescence intensity quantification in the gray matter is presented in the lower panels. Color coding: GFAP, magenta; NeuN, green. Scale bars, 400 µm (**left**) and 100 µm (**right**). (**B**) Iba1 immunofluorescence analysis in lumbar spinal cord sections from SOD1 mice receiving the indicated rAAV2-retro vectors. Representative images are shown in the upper panels, and the lower panels provide quantitative measurements of Iba1 fluorescence in the gray matter. Color coding: Iba1, magenta; NeuN, green; DAPI, blue. Scale bars, 400 µm (**left**) and 100 µm (**right**). Data are means ± SEM, *n* = 5 mice per group. Multiple ROIs per animal were averaged to generate a single value per mouse before statistical analysis. One-way ANOVA with Tukey’s post hoc test. * *p* < 0.05, ** *p* < 0.01, *** *p* < 0.001.

Microglial and astrocytic activation is a hallmark of ALS pathology. Both the miRNA-alone and combination vectors significantly lowered Iba1 (microglia) and GFAP (astrocyte) fluorescence in the spinal cord gray matter (Figure 8; Table 1). IGF1 alone changed neither marker relative to scrambles (Table 1), and the miRNA-alone and combination groups did not differ in this respect (Table 1).

### 3.7. miRNA-Mediated hSOD1 Knockdown Reduces Protein Accumulation and Spinal Cord Apoptosis

**Figure 9:**
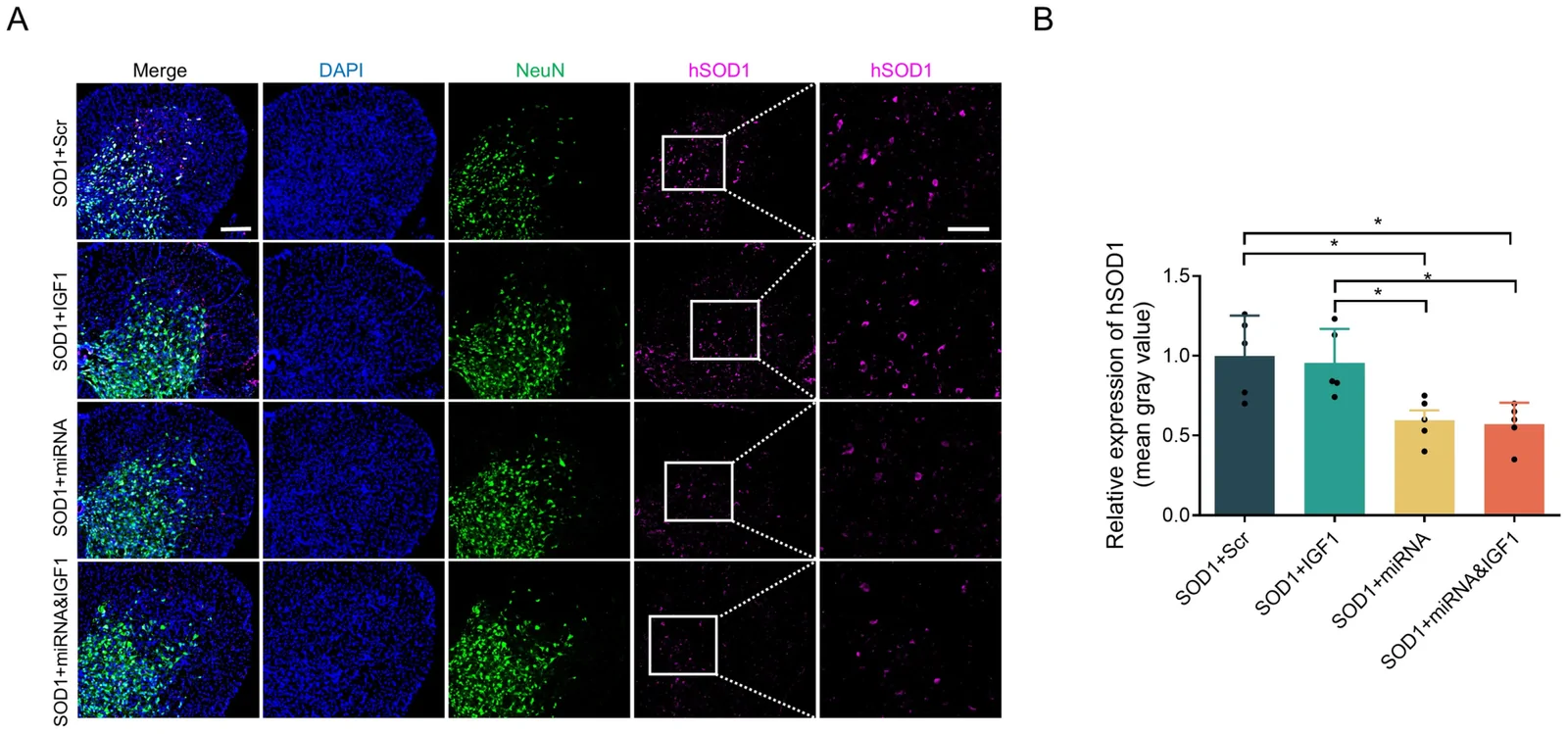
hSOD1 protein accumulation in the spinal cord of SOD1 mice following intrathecal delivery of rAAV2-retro-based vectors. (**A**) Lumbar spinal cord hSOD1 immunofluorescence in SOD1 mice receiving the indicated rAAV2-retro vectors. hSOD1 immunoreactivity was predominantly distributed in the cytoplasm of neurons. Color coding: hSOD1, magenta; NeuN, green; DAPI, blue. Scale bar, 200 µm and 100 µm. (**B**) Quantification of *hSOD1* signal in lumbar spinal cord regions. Data are means ± SEM; *n* = 5 mice per group. Multiple ROIs per animal were averaged to generate a single value per mouse before statistical analysis. One-way ANOVA with Tukey’s post hoc test. * *p* < 0.05.

**Figure 10:**
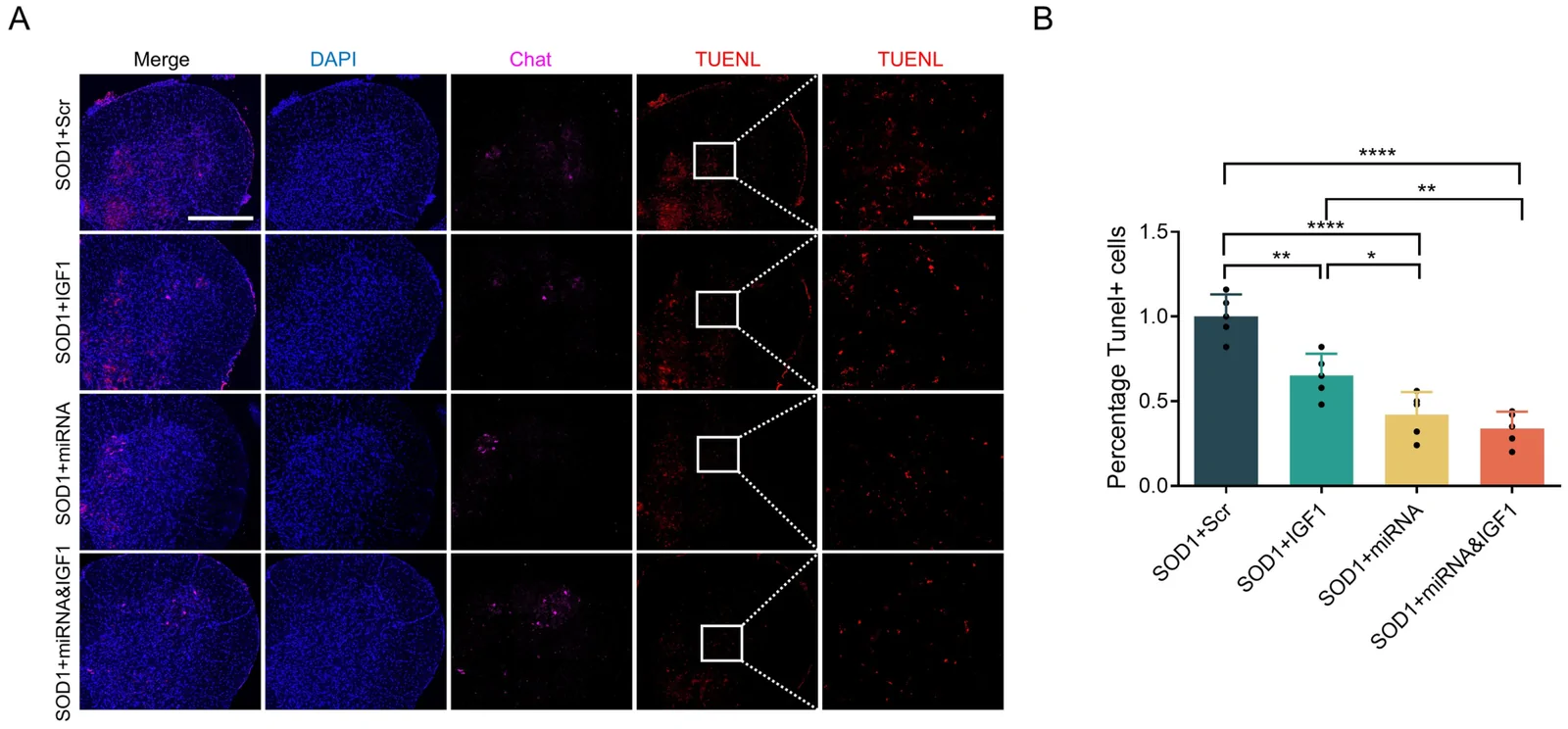
Assessment of spinal cord cell apoptosis in SOD1 mice following intrathecal delivery of rAAV2-retro-based vectors. (**A**) Representative images showing TUNEL staining in lumbar spinal cord sections from SOD1 mice receiving the indicated rAAV2-retro vectors. Color coding: TUNEL, red; ChAT, magenta; DAPI, blue. Scale bars, 400 µm and 100 µm. (**B**) TUNEL-positive cell counts in the lumbar spinal cord. Data are means ± SEM; *n* = 5 mice per group. One-way ANOVA with Tukey’s post hoc test. Multiple spinal cord sections per animal were averaged to generate a single value per mouse before statistical analysis. * *p* < 0.05, ** *p* < 0.01, **** *p* < 0.0001.

At day 130, hSOD1 immunoreactivity was still markedly reduced in the spinal cord of mice treated with the miRNA-alone or combination vectors, whereas IGF1 alone had no effect (Figure 9; Table 1). TUNEL staining detects DNA fragmentation and thus marks dying cells, showing that the IGF1-alone, miRNA-alone, and combination treatments all lowered TUNEL-positive cell counts in the spinal cord relative to the controls (Figure 10A,B; Table 1). The effect was strongest for the miRNA-alone and combination vectors (*p* < 0.0001) and weaker, though still clear, for IGF1 alone (*p* < 0.01). The miRNA-alone and combination groups did not differ in *hSOD1* reduction or TUNEL-positive cell counts (Table 1).

For most endpoints, the 95% confidence intervals for the miRNA vs. combination comparison included the null value (Table 1).

## 4. Discussion

For the first time, we directly compared the effects of intrathecal rAAV2-retro-mediated *hSOD1* silencing, *IGF1* overexpression, and their combination in adult *SOD1*^G93A^ mice. miRNA-mediated *hSOD1* silencing significantly lengthened median survival (from 139.5 days in controls to 164.5 days in the miRNA-alone group, +25 days), improved motor function (forelimb and hindlimb grip strength and rotarod latency), protected neuromuscular junctions, limited spinal α-motor neuron loss, and damped gliosis and apoptosis. IGF1 alone also increased survival (median 152 days, +12.5 days) and spared neurons (significant preservation of ChAT^+^ neurons), yet it left grip strength and rotarod performance unchanged, prolonging life without preserving function. Contrary to expectations, adding IGF1 to the miRNA did not result in a statistically significant improvement over miRNA alone on any outcome measure. The dominant contributor in this model is therefore miRNA-mediated *hSOD1* silencing; IGF1, effective on its own, did not provide a statistically detectable additional benefit on top of hSOD1 silencing under these conditions.

The vector itself performed well as a gene-delivery vehicle. One intrathecal dose of rAAV2-retro-CAG-GFP reached every spinal cord segment (cervical, thoracic, and lumbar), as well as brain regions, including the motor cortex and brainstem. Almost all GFP-positive cells in the lumbar cord were ChAT-positive, indicating strong motor neuron tropism. AAV9 administered by the same route transduced the liver and heart far more heavily. That difference may be relevant for a single-dose gene delivery approach, although comprehensive systemic biodistribution and toxicological assessments were not performed. Overall transduction intensity across spinal segments matched AAV9, with lumbar enrichment, which makes rAAV2-retro a practical and efficient gene-delivery platform in this model; whether these properties translate to other disease contexts remains to be determined. The therapeutic benefits observed in the present study using intrathecal delivery in adult mice extend our previous findings in neonatal mice, where intramuscular injection was used [29]. Together, these two studies establish rAAV2-retro as a versatile platform for motor neuron-targeted gene delivery, with the current work providing a clinically more translatable route of administration. We then used it to test miRNA-mediated *hSOD1* silencing and *IGF1* overexpression.

Several hypotheses may account for the absence of an additive effect of IGF1 when combined with *hSOD1* silencing. First, the two interventions may converge on shared downstream effectors, such as the Bcl-2 family and the caspase cascade; if *hSOD1* silencing alone already saturates these pathways, additional IGF1 input would add little. Second, the dose and exposure of IGF1 achieved by intrathecal delivery in this study may be insufficient to produce a measurable additive effect. Third, the therapeutic window may be a critical factor: IGF1 may be more effective at earlier, pre-symptomatic stages, as suggested by the neonatal treatment paradigm used by Baird et al. [42], whereas at 8 weeks of age, the neuroinflammatory milieu is already established. Fourth, a ceiling effect may exist, whereby *hSOD1* silencing alone already maximally engages neuronal survival pathways, leaving little room for additional neurotrophic support. Fifth, this study was not powered to detect small additive effects, and a modest benefit of IGF1 cannot be excluded. These hypotheses are not mutually exclusive and require further experimental validation. Note that these conclusions are based on a single intrathecal administration. Whether repeated dosing could produce additional benefit remains unknown, but repeated administration of the same AAV serotype is constrained by the induction of anti-capsid neutralizing antibodies, which prevent re-transduction by the same serotype [43]. Alternative strategies, such as using a different capsid for subsequent doses, would require further investigation.

Three practical points follow for gene therapy in *SOD1*-ALS. First, miRNA-mediated hSOD1 silencing showed therapeutic efficacy in this *SOD1^G93A^* mouse model: longer survival, better motor performance, and fewer lost motor neurons all followed from the injection of rAAV2-retro-miRNA. Whether this approach is effective in human *SOD1*-ALS remains to be established. Second, the rAAV2-retro platform provides a one-dose delivery mode in this model. Compared with tofersen, which is a recently approved antisense oligonucleotide that must be re-injected via lumbar puncture, an AAV-delivered miRNA may offer the advantage of sustained expression from a single administration, avoiding the need for repeated lumbar punctures. The mechanistic differences between AAV-delivered miRNA and antisense oligonucleotides (ASOs) also merit consideration. ASOs act through RNase H-mediated mRNA degradation and require repeated administration to maintain target suppression, whereas AAV-delivered miRNA provides sustained silencing from a single dose. This distinction is clinically relevant: tofersen requires lifelong intrathecal dosing via lumbar puncture, while a one-time AAV injection could achieve durable SOD1 suppression. The clinical feasibility of this strategy was initially explored in the Phase 1/2 EPISOD1 trial of AMT-162, which delivered an anti-SOD1 miRNA via a single intrathecal injection using an AAVrh10 capsid. Development of AMT-162 was subsequently discontinued following a dose-limiting dorsal root ganglia toxicity, underscoring that vector-specific biodistribution and dose optimization remain critical for clinical translation [44]. In our study, rAAV2-retro was selected for its enhanced retrograde transport and motor neuron tropism, which resulted in widespread spinal cord transduction with limited expression in the liver and heart, the only peripheral organs examined. Whether these capsid properties translate into improved clinical outcomes compared with AAVrh10 remains to be determined. Note that AAV-mediated silencing is not readily reversible or dose-adjustable, unlike ASO therapy, which is an important consideration for clinical translation if excessive target suppression or immune responses arise. Third, rAAV2-retro barely transduced the liver and heart, far less than AAV9 (Figure S2), which may reduce the potential for liver-related off-target effects, although we did not directly assess hepatotoxicity in this study. IGF1 alone did extend survival and protect neurons here, but adding it to *hSOD1* silencing did not significantly improve outcomes over miRNA alone. The one exception was a modest, nominally significant improvement in hindlimb clasping; given the number of comparisons performed, this finding should be interpreted as exploratory and requires confirmation in adequately powered studies. Given the present evidence, no statistically detectable additional benefit of adding IGF1 to *hSOD1* silencing was observed when treatment started intrathecally at 8 weeks under the conditions tested; whether other regimens, doses, or earlier treatment windows might show additive effects remains to be determined. Other regimens, higher doses, earlier treatment at neonatal or pre-symptomatic stages, or different *IGF1* isoforms (such as IGF1-Ea or mechano-growth factor) may still prove additive and are worth testing. What this study does establish is that rAAV2-retro-delivered miRNA silencing of *hSOD1* is effective in this *SOD1^G93A^* mouse model, but no statistically detectable additional benefit of IGF1 was observed under the conditions tested here.

Several limitations of this study should be acknowledged. First, peripheral transduction was assessed only in the liver and heart, and these assessments were performed in C57BL/6J mice rather than in the *SOD1^G93A^* model; the observed distribution profile should therefore be interpreted as potentially favorable rather than as demonstrated safety, and a more comprehensive biodistribution analysis across additional organs and in the *SOD1^G93A^* model will be required. Second, disease onset was defined as a 10% decline from peak body weight. Although this definition is precedented in *SOD1^G93A^* studies, it is not equivalent to motor onset and may be influenced by treatment-related metabolic effects, particularly given that IGF1 was one of the interventions tested. IGF1 has known anabolic and metabolic actions that could independently affect body weight [45,46]. Our behavioral assessments were performed weekly, which precluded a reliable motor-based onset definition. Therefore, the onset data should be interpreted with caution, and future studies with more frequent behavioral monitoring and a motor-based onset criterion would be needed to confirm the temporal pattern of treatment effects. Third, the study was not prospectively powered to demonstrate equivalence or non-inferiority between the miRNA and combination groups, and the group sizes were modest. For most endpoints, the 95% confidence intervals for the miRNA vs. combination comparison included zero, indicating that the absence of a statistically significant difference should not be interpreted as definitive evidence of equivalence. Rather, no statistically detectable additional benefit of IGF1 was observed under the conditions tested. Fourth, a full longitudinal mixed-effects model was not used because the missing data at late time points were not missing at random (they resulted from disease progression and humane endpoints), which limits the validity of conventional longitudinal inference. Cross-sectional analyses were performed instead, which avoid the bias of non-random missingness but do not model within-animal correlation across the full time course. Fifth, both male and female mice were included in this study, but it was not powered to detect sex-specific effects. Although sex-related differences in disease phenotype have been reported in the *SOD1^G93A^* model, these effects are strain-dependent; against the C57BL/6J background used here, no significant sex differences in survival or disease duration were observed [47]. Future studies with larger cohorts are needed to formally assess whether treatment responses differ by sex. Sixth, this study assessed multiple endpoints, which increases the risk of type-I error across the broader family of endpoints. Survival analysis was pre-specified as the principal confirmatory endpoint; all other endpoints were analyzed as exploratory. The *p* values for exploratory endpoints should therefore be interpreted as hypothesis-generating rather than confirmatory, and future studies with a pre-specified primary endpoint and formal multiplicity control are needed to confirm these findings. The week-19 behavioral comparison was a cross-sectional analysis at the last common time point, consistent with the approach used in prior *SOD1*-related ALS studies [16]. It does not capture the treatment × time trajectory, and the longitudinal structure of the behavioral data was not modeled. In addition, the number of measurable animals per group at week 19 was small, and these results should be interpreted with caution. Seventh, WT littermates were not included in the histological analysis of motor neuron counts, so the comparison was restricted to treatment groups. Eighth, the *SOD1*^G93A^ model is a specific and aggressive genetic model that does not capture the molecular heterogeneity of sporadic ALS. Our findings are therefore most directly relevant to SOD1-associated ALS, and their generalizability to the broader ALS spectrum remains to be established. Ninth, several mechanisms have been proposed to explain why no statistically detectable additional benefit of IGF1 was observed. These include saturation of shared downstream survival pathways, insufficient IGF1 exposure, a narrow intervention window, ceiling effects from *hSOD1* silencing, and limited statistical power. All remain hypotheses that require further experimental validation.

Beyond these limitations, several avenues warrant further investigation. miRNA-mediated *hSOD1* silencing works on its own in *SOD1*-ALS, but there is room to push efficacy further and to broaden translational applicability. The *IGF1* cassette itself could be improved. IGF1 alone extended survival here, but the effect was small and did not synergize with *hSOD1* silencing. A stronger promoter, such as Synapsin, would give higher, more sustained *IGF1* expression in motor neurons. In addition, fusion tags, such as the Fc fragment of immunoglobulin or a cell-penetrating peptide, might improve its secretion, stability, and spread through the CNS and, thus, its paracrine reach into neighboring neurons and glia. Another question is whether a different neurotrophic factor would do better. The absence of an additive effect in this study applies specifically to IGF1 under the conditions tested and should not be extrapolated to all neurotrophic factors. IGF1 did not provide a measurable additional benefit here, so factors acting through other pathways deserve a look. Glial cell line-derived neurotrophic factor (GDNF) [2,48] and brain-derived neurotrophic factor (BDNF) [49] both protect motor neurons in preclinical ALS models and signal through Ret and TrkB, which only partly overlap the PI3K/Akt route used by IGF1. Pairing *hSOD1* silencing with GDNF or BDNF might therefore add what IGF1 could not, removing the toxic protein while supporting neuronal function by a separate route. A single rAAV2-retro genome could carry two or three such cassettes. Dose-escalation studies are also needed to fix the optimal vector dose, and long-term safety work in large animals will be required before rAAV2-retro-based platforms can be considered for further translational development in *SOD1*-ALS. Whether the approach is generalizable to other motor neuron diseases remains to be established.

## Figures and Tables

**Table 1:** Statistical summary of pairwise comparisons across phenotypic measures among treatment groups.

Phenotype		SOD1 + Scr vs. SOD1 + IGF1	SOD1 + Scr vs. SOD1 + miRNA	SOD1 + Scr vs. SOD1 + miRNA & IGF1	SOD1 + IGF1 vs. SOD1 + miRNA	SOD1 + IGF1 vs. SOD1 + miRNA & IGF1	SOD1 + miRNA vs. SOD1 + miRNA & IGF1
Disease onset		*p* = 0.9144 Cohen’s d = 0.35 95.00% Cl −20.95~12.78	*p* = 0.0128 Cohen’s d = 1.87 95.00% Cl −37.20~−3.470	*p* = 0.0002 Cohen’s d = 1.98 95.00% Cl −41.05~−11.47	*p* = 0.1017 Cohen’s d = 1.14 95.00% Cl −34.72~2.223	*p* = 0.005 Cohen’s d = 1.53 95.00% Cl −38.78~−5.571	*p* = 0.7729 Cohen’s d = 0.38 95.00% Cl −22.53~10.68
Median Survival		*p* = 0.0161 HR = 2.558 95.00% Cl 1.032~6.343	*p* = 0.0004 HR = 5.214 95.00% Cl 1.876~14.49	*p* <0.0001 HR = 8.326 95.00% Cl 2.874~24.12	*p* = 0.0312 HR = 2.184 95.00% Cl 1.063~4.487	*p* = 0.0058 HR = 3.127 95.00% Cl 1.372~7.128	*p* = 0.6842 HR = 1.214 95.00% Cl 0.478~3.082
Grip strength	Forelimb	*p* = 0.5728 Cohen’s d = 0.7 95.00% Cl −48.37~ 17.36	*p* = 0.009 Cohen’s d = 1.54 95.00% Cl −74.67~−8.948	*p* = 0.0002 Cohen’s d = 3.26 95.00% Cl −80.72~−22.94	*p* = 0.1229 Cohen’s d = 0.94 95.00% Cl −57.64~5.028	*p* = 0.0058 Cohen’s d = 2.06 95.00% Cl 9.189~ 63.46	*p* = 0.742 Cohen’s d = 0.41 95.00% Cl −17.12~37.15
	Hindlimb	*p* = 0.5181 Cohen’s d = 0.93 95.00% Cl −59.25~19.50	*p* = 0.0364 Cohen’s d = 2.29 95.00% Cl −77.40~−1.994	*p* = 0.0066 Cohen’s d = 1.94 95.00% Cl −75.96~−10.43	*p* = 0.4837 Cohen’s d = 0.83 95.00% Cl −57.52~17.88	*p* = 0.2306 Cohen’s d = 0.88 95.00% Cl −9.444~56.09	*p* = 0.9891 Cohen’s d = 0.15 95.00% Cl −27.23~34.23
Rotarod		*p* = 0.6948 Cohen’s d = 0.75 95.00% Cl −57.20~24.43	*p* = 0.0178 Cohen’s d = 1.67 95.00% Cl −88.32~−6.691	*p* = 0.0025 Cohen’s d = 2.1 95.00% Cl −89.84~−16.47	*p* = 0.1269 Cohen’s d = 1.07 95.00% Cl −68.38~6.139	*p* = 0.0229 Cohen’s d = 1.4 95.00% Cl 4.091~69.45	*p* = 0.9646 Cohen’s d = 0.2 95.00% Cl −27.03~38.33
Limb clasping score		*p* > 0.9999 VDA = 0.523 95.00% Cl 0.253~0.794	*p* = 0.5297 VDA = 0.609 95.00% Cl 0.323~0.896	*p* = 0.0026 VDA = 0.808 95.00% Cl 0.585~1.000	*p* > 0.9999 VDA = 0.586 95.00% Cl 0.266~0.906	*p* = 0.1643 VDA = 0.827 95.00% Cl 0.558~1.000	*p* = 0.9668 VDA = 0.74 95.00% Cl 0.388~1.000
Endplate Innervation	0%	*p* = 0.2437 Cohen’s d = 1.83 95.00% Cl −2.761~14.76	*p* < 0.0001 Cohen’s d = 8.25 95.00% Cl 18.24~35.76	*p* < 0.0001 Cohen’s d = 9.16 95.00% Cl 21.24 ~ 38.76	*p* < 0.0001 Cohen’s d = 6.41 95.00% Cl 12.24~29.76	*p* < 0.0001 Cohen’s d = 7.33 95.00% Cl 15.24~32.76	*p* = 0.7627 Cohen’s d = 0.92 95.00% Cl −5.761~11.76
	<50%	*p* = 0.0532 Cohen’s d = 2.54 95.00% Cl −12.07~0.06914	*p* = 0.0031 Cohen’s d = 3.81 95.00% Cl −15.07~−2.931	*p* = 0.2727 Cohen’s d = 1.69 95.00% Cl −10.07~2.069	*p* = 0.509 Cohen’s d = 1.27 95.00% Cl −9.069~3.069	*p* = 0.7826 Cohen’s d = 0.85 95.00% Cl −4.069~8.069	*p* = 0.1264 Cohen’s d = 2.12 95.00% Cl −1.069~11.07
	>50%	*p* = 0.4266 Cohen’s d =1.41 95.00% Cl −3.329~11.33	*p* = 0.862 Cohen’s d =0.71 95.00% Cl −9.329~5.329	*p* = 0.1297 Cohen’s d =2.12 95.00% Cl −13.33~1.329	*p* = 0.1297 Cohen’s d =2.12 95.00% Cl −13.33~1.329	*p* = 0.0062 Cohen’s d =3.53 95.00% Cl −17.33~−2.671	*p* = 0.4266 Cohen’s d =1.41 95.00% Cl −11.33~3.329
	100%	*p* = 0.398 Cohen’s d = 1.44 95.00% Cl −11.08~3.083	*p* < 0.0001 Cohen’s d = 5.78 95.00% Cl −23.08~−8.917	*p* < 0.0001 Cohen’s d = 7.22 95.00% Cl −27.08~−12.92	*p* = 0.0009 Cohen’s d = 4.33 95.00% Cl −19.08~−4.917	*p* < 0.0001 Cohen’s d = 5.78 95.00% Cl −23.08~−8.917	*p* = 0.398 Cohen’s d = 1.44 95.00% Cl −11.08~3.083
Average fiber area		*p* = 0.0337 Cohen’s d = 2.74 95.00% Cl −692.6~−23.69	*p* < 0.0001 Cohen’s d = 7.18 95.00% Cl −1273~−604.0	*p* < 0.0001 Cohen’s d = 8.55 95.00% Cl −1453~−783.7	*p* = 0.0007 Cohen’s d = 4.44 95.00% Cl −914.8~−245.9	*p* < 0.0001 Cohen’s d = 5.81 95.00% Cl −1094~−425.5	*p* = 0.4718 Cohen’s d = 1.37 95.00% Cl −514.1~154.8
CMAP		*p* = 0.6659 Cohen’s d = 0.87 95.00% Cl −4.398~1.878	*p* = 0.0196 Cohen’s d = 2.35 95.00% Cl −6.798~−0.5223	*p* = 0.003 Cohen’s d = 2.58 95.00% Cl −7.807~−1.532	*p* = 0.1687 Cohen’s d = 1.45 95.00% Cl −5.538~0.7377	*p* = 0.0309 Cohen’s d = 1.8 95.00% Cl −6.547~−0.2721	*p* = 0.7943 Cohen’s d = 0.51 95.00% Cl −4.147~2.128
ChAT+ cells		*p* = 0.0363 Cohen’s d = 2.67 95.00% Cl −1.221~−0.038	*p* = 0.0003 Cohen’s d = 3.51 95.00% Cl −1.950~−0.4947	*p* = 0.0001 Cohen’s d = 2.86 95.00% Cl −2.064~−0.6087	*p* = 0.0495 Cohen’s d = 1.75 95.00% Cl −1.320~0.1350	*p* = 0.0175 Cohen’s d = 1.53 95.00% Cl −1.434~0.02089	*p* = 0.9427 Cohen’s d = 0.22 95.00% Cl −0.8416~0.6135
GFAP fluorescence intensity		*p* = 0.3699 Cohen’s d = 1.06 95.00% Cl −0.07890~0.3005	*p* = 0.0004 Cohen’s d = 3.36 95.00% Cl 0.1623~ 0.5417	*p* = 0.0001 Cohen’s d = 3.7 95.00% Cl 0.1983~0.5777	*p* = 0.0107 Cohen’s d = 2.3 95.00% Cl 0.05147~0.4309	*p* = 0.0036 Cohen’s d = 2.64 95.00% Cl 0.08747~0.4669	*p* = 0.9471 Cohen’s d = 0.34 95.00% Cl −0.1537~0.2257
Iba1 fluorescence intensity		*p* = 0.8782 Cohen’s d = 0.66 95.00% Cl −0.1351~0.2299	*p* = 0.0002 Cohen’s d = 5.16 95.00% Cl 0.1855~0.5505	*p* = 0.0001 Cohen’s d = 5.28 95.00% Cl 0.1941~0.5591	*p* = 0.0006 Cohen’s d = 4.5 95.00% Cl 0.1381~0.5031	*p* = 0.0005 Cohen’s d = 4.62 95.00% Cl 0.1467~ 0.5117	*p* = 0.9992 Cohen’s d = 0.12 95.00% Cl −0.1739~0.1911
hSOD1 expression levels		*p* = 0.982 Cohen’s d = 0.15 95.00% Cl −0.3012~ 0.3908	*p* = 0.0195 Cohen’s d = 1.46 95.00% Cl 0.05801~0.7500	*p* = 0.0126 Cohen’s d = 1.32 95.00% Cl 0.08401~ 0.7760	*p* = 0.0405 Cohen’s d = 1.3 95.00% Cl 0.01320~0.7052	*p* = 0.0266 Cohen’s d = 1.18 95.00% Cl 0.03920~0.7312	*p* = 0.9963 Cohen’s d = 0.09 95.00% Cl −0.3200~0.3720
TUNEL+ cells		*p* = 0.002 Cohen’s d = 2.93 95.00% Cl 0.1251~0.5749	*p* < 0.0001 Cohen’s d = 4.86 95.00% Cl 0.3551~0.8049	*p* < 0.0001 Cohen’s d = 5.54 95.00% Cl 0.4371~0.8869	*p* = 0.0442 Cohen’s d = 1.93 95.00% Cl 0.005050~0.4549	*p* = 0.0055 Cohen’s d = 2.61 95.00% Cl 0.08705~0.5369	*p* = 0.7275 Cohen’s d = 0.69 95.00% Cl −0.1429~0.3069

Statistical summary of pairwise comparisons across phenotypic measures among treatment groups. Survival data were analyzed using Kaplan–Meier curves and compared using the log-rank (Mantel–Cox) test. Forelimb and hindlimb grip strength and rotarod performance were analyzed at week 19 using one-way ANOVA followed by Tukey’s post hoc test. Limb clasping scores were analyzed using the Kruskal–Wallis test followed by Dunn’s post hoc test. Molecular and histological data (ChAT+ cells, GFAP fluorescence intensity, Iba1 fluorescence intensity, hSOD1 expression levels, and TUNEL+ cells) were analyzed using one-way ANOVA followed by Tukey’s post hoc test. Exact *p* values are reported for all pairwise comparisons. ChAT+ cells, number of ChAT-positive motor neurons; GFAP fluorescence intensity, mean GFAP fluorescence intensity in the gray matter; Iba1 fluorescence intensity, mean Iba1 fluorescence intensity in the gray matter; hSOD1 expression levels, relative hSOD1 protein levels; TUNEL+ cells, number of TUNEL-positive cells. ns, not significant.

## Data Availability

The original data generated and/or analyzed in this study are available within the article and its Supplementary Material. Additional information can be obtained from the corresponding authors upon reasonable request.

## Supplementary Materials

The following supporting information can be downloaded at https://www.mdpi.com/article/10.3390/cells15201890/s1, Figure S1: Quantification of eGFP and ChAT co-localization across spinal cord segments. Figure S2: Assessment of peripheral organ transduction after intrathecal delivery of rAAV2-retro and AAV9. Figure S3: Assessment of transduction in different spinal cord segments following intrathecal injection of rAAV2-retro and AAV9.
