# Supplementary material for: Single-Dose Intrathecal rAAV2-Retro Delivering Anti-hSOD1 miRNA Confers Robust Neuroprotection in Adult ALS Mice
Source: Cells. 2026 Oct 16;15(20):1890. doi: 10.3390/cells15201890 (PMC13703363; doi:10.3390/cells15201890)
Supplement: Supplementary file 1 [file cells-15-01890-s001.zip › cells-4541947-supplementary.pdf]

Supplemental Information

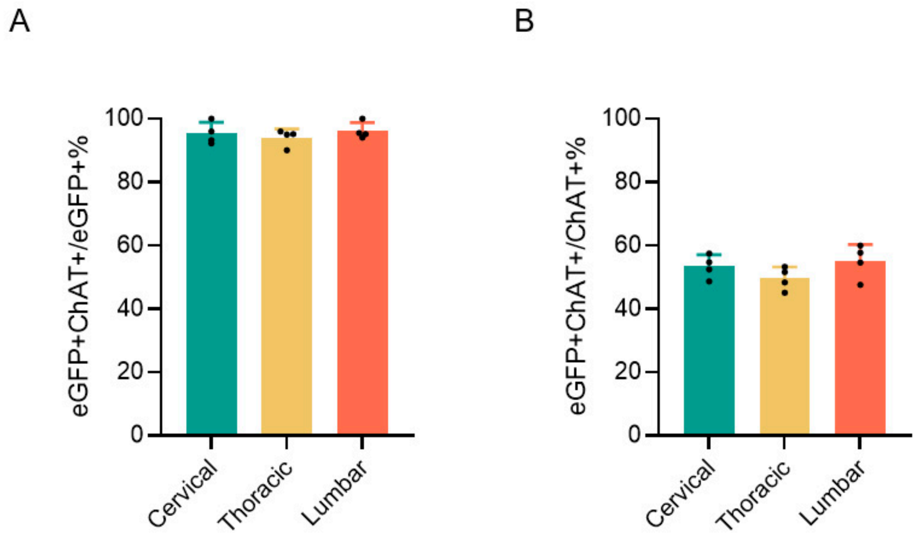

Figure S1. Quantification of eGFP and ChAT co-localization across spinal cord segments. (A) Specificity: proportion of eGFP-positive cells that were ChAT-positive in the cervical, thoracic, and lumbar segments. (B) Coverage: proportion of ChAT-positive motor neurons that were eGFP-positive in the cervical, thoracic, and lumbar segments. Data are means ± SEM (n = 4 mice per segment).

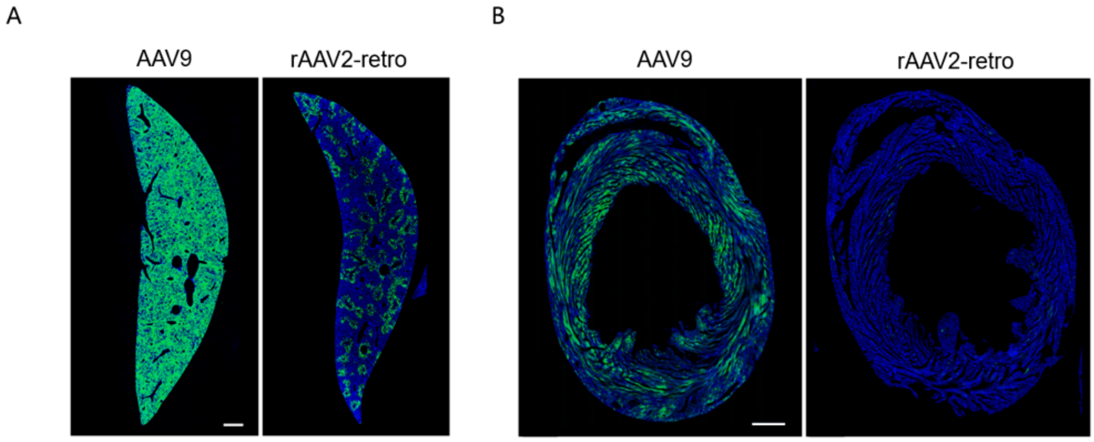

Figure S2. Assessment of liver and heart transduction after intrathecal delivery of rAAV2-retro and AAV9. (A) Representative images of liver sections from mice given intrathecal rAAV2-retro or AAV9. Scale bar, 500 μm. (B) Representative images of heart sections from mice given intrathecal rAAV2-retro or AAV9. Scale bar, 500 μm.

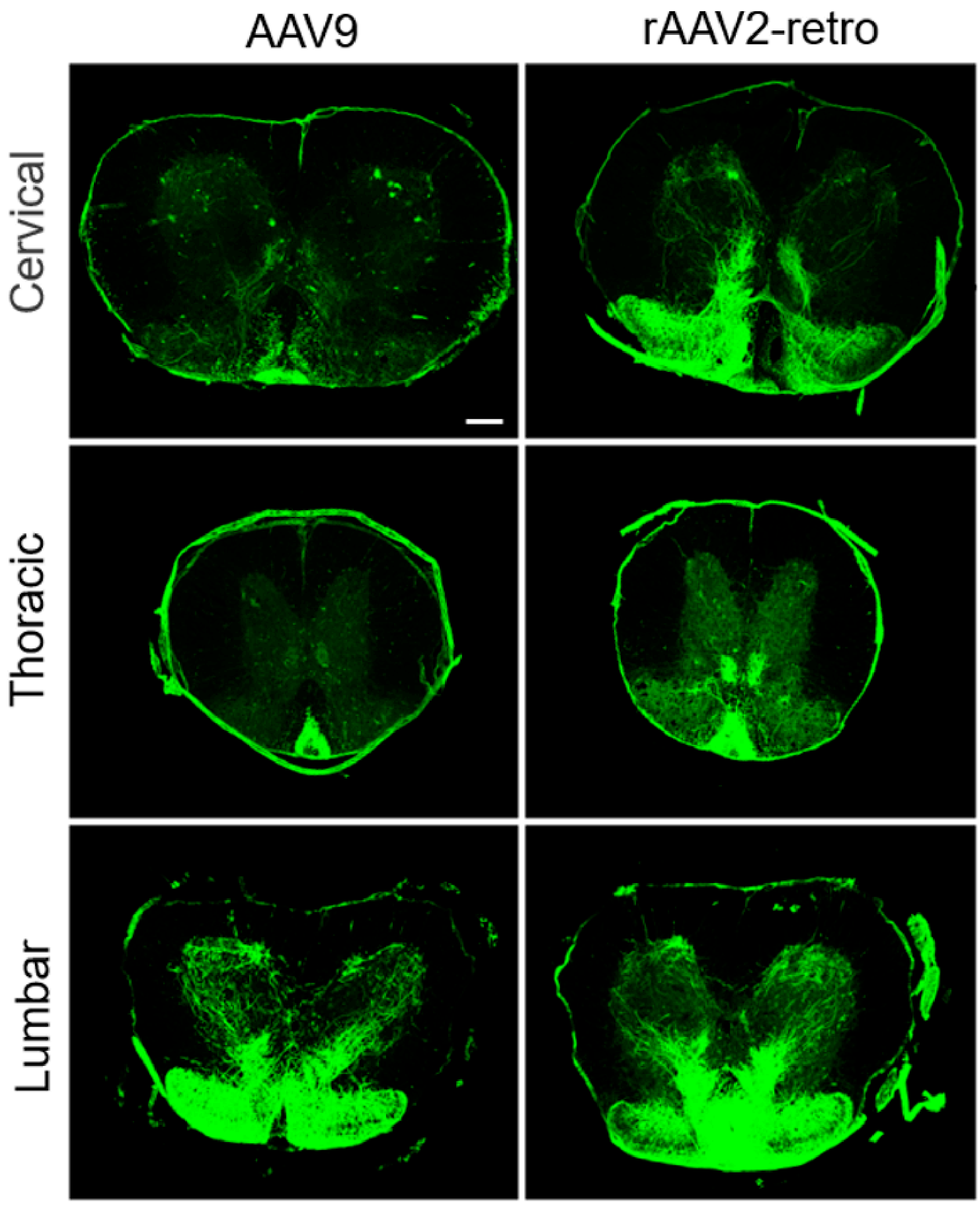

Figure S3. Assessment of transduction in different spinal cord segments following intrathecal injection of rAAV2-retro and AAV9. Representative spinal cord sections from mice given intrathecal rAAV2-retro or AAV9. Scale bar, 200 μm.
